# Glucagon-like peptide-1 receptor: mechanisms and advances in therapy

**DOI:** 10.1038/s41392-024-01931-z

**Published:** 2024-09-18

**Authors:** Zhikai Zheng, Yao Zong, Yiyang Ma, Yucheng Tian, Yidan Pang, Changqing Zhang, Junjie Gao

**Affiliations:** 1 Department of Orthopaedics, Shanghai Sixth People’s Hospital Affiliated to Shanghai Jiao Tong University School of Medicine, Shanghai, 200233 China; 2 Institute of Microsurgery on Extremities, and Department of Orthopedic Surgery, Shanghai Sixth People’s Hospital Affiliated to Shanghai Jiao Tong University School of Medicine, Shanghai, 200233 China; 3 Centre for Orthopaedic Research, Medical School, The University of Western Australia, Nedlands, WA 6009 Australia

**Keywords:** Metabolic disorders, Cell biology

## Abstract

The glucagon-like peptide-1 (GLP-1) receptor, known as GLP-1R, is a vital component of the G protein-coupled receptor (GPCR) family and is found primarily on the surfaces of various cell types within the human body. This receptor specifically interacts with GLP-1, a key hormone that plays an integral role in regulating blood glucose levels, lipid metabolism, and several other crucial biological functions. In recent years, GLP-1 medications have become a focal point in the medical community due to their innovative treatment mechanisms, significant therapeutic efficacy, and broad development prospects. This article thoroughly traces the developmental milestones of GLP-1 drugs, from their initial discovery to their clinical application, detailing the evolution of diverse GLP-1 medications along with their distinct pharmacological properties. Additionally, this paper explores the potential applications of GLP-1 receptor agonists (GLP-1RAs) in fields such as neuroprotection, anti-infection measures, the reduction of various types of inflammation, and the enhancement of cardiovascular function. It provides an in-depth assessment of the effectiveness of GLP-1RAs across multiple body systems-including the nervous, cardiovascular, musculoskeletal, and digestive systems. This includes integrating the latest clinical trial data and delving into potential signaling pathways and pharmacological mechanisms. The primary goal of this article is to emphasize the extensive benefits of using GLP-1RAs in treating a broad spectrum of diseases, such as obesity, cardiovascular diseases, non-alcoholic fatty liver disease (NAFLD), neurodegenerative diseases, musculoskeletal inflammation, and various forms of cancer. The ongoing development of new indications for GLP-1 drugs offers promising prospects for further expanding therapeutic interventions, showcasing their significant potential in the medical field.

## Introduction

In recent years, GLP-1R and its agonists have garnered widespread attention in the medical community. GLP-1R, a core member of the GPCR family, is widely present on the surfaces of various cells in the human body.^1,2^ By specifically binding to the key hormone GLP-1, it regulates blood glucose levels and lipid metabolism.^3,4^ This receptor and its agonists hold significant therapeutic potential, reshaping the treatment approaches for multiple diseases, including diabetes, cardiovascular disorders, and neurodegenerative diseases.^5–7^ GLP-1 is a peptide produced by the cleavage of proglucagon, mainly synthesized in the intestinal mucosal L-cells, pancreatic islet α-cells, and neurons in the nucleus of the solitary tract.^3,4^ GLP-1RAs mimic the action of endogenous GLP-1, activating GLP-1R, thereby enhancing insulin secretion, inhibiting glucagon release, delaying gastric emptying, and reducing food intake through central appetite suppression.^8–10^ These mechanisms make GLP-1RAs powerful tools for controlling blood glucose and improving metabolic syndrome. Furthermore, their multifaceted mechanisms of action suggest potential applications beyond traditional metabolic disorders. From the discovery of the GLP-1 fragment GLP-1(7-37) to the development of more stable and long-acting GLP-1 analogs, these milestones represent significant breakthroughs in the medical field.^11,12^ For instance, the success of exenatide has not only spurred the development of potent GLP-1 analogs such as liraglutide and semaglutide but also unveiled the vast potential of GLP-1RAs in treating various systemic diseases. These developments underscore the importance of GLP-1RAs in modern therapeutics. The applications of GLP-1RAs extend far beyond diabetes management.^5^

Here we summarized the complex mechanisms of GLP-1RAs and their latest advancements in treating various diseases, such as musculoskeletal inflammation, obesity, cardiovascular diseases, NAFLD, neurodegenerative diseases, and various cancers. We introduce recent studies that demonstrate the remarkable performance of GLP-1RAs in slowing the progression of neurodegenerative diseases, reducing inflammation, and enhancing cardiovascular health. For example, in the treatment of Alzheimer’s diseases (AD) and Parkinson’s diseases (PD), GLP-1RAs have shown potential in slowing disease progression, while their anti-inflammatory properties offer new hope for conditions such as osteoarthritis (OA), rheumatoid arthritis (RA) and cardiovascular diseases.^13–15^ By integrating the latest clinical trial data, we explore the efficacy of GLP-1RAs in treating diseases of the nervous, cardiovascular, endocrine, and digestive systems. We show readers that GLP-1RAs have also been found to significantly reduce the risks of heart failure, atherosclerosis (AS), and hypertension, highlighting their broad therapeutic potential.^16–19^ As new indications continue to be developed, GLP-1 drugs demonstrate immense potential in the medical field, with future research expected to expand their therapeutic applications. The comprehensive exploration of their benefits underscores their transformative potential in medicine, positioning them as a promising approach for addressing a wide array of health issues and paving the way for new research and clinical applications.

The future of GLP-1RA therapy is promising. Researchers are developing more efficient formulations, such as long-acting and oral versions, to improve patient compliance and outcomes. With increasing clinical evidence, GLP-1RAs are set to become essential in treating complex and chronic diseases, offering significant health benefits and addressing unmet medical needs, thus enhancing patient quality of life and solidifying their role in future medical advancements.

## Review of the history and milestones in GLP-1 research

**Fig. 1:**
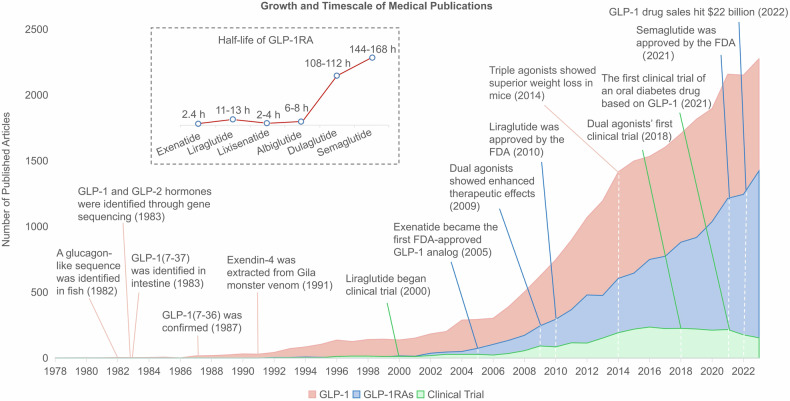
Growth and Timescale of Medical Publications on GLP-1 and GLP-1RAs. This figure illustrates the number of published articles over time, from 1978 to 2022, related to GLP-1 and GLP-1RAs, along with significant milestones in clinical trials. The data is visualized through three layered area graphs, each representing a different category of publications: research on GLP-1 (red area), GLP-1RAs (blue area), and clinical trials (green area). Key milestones are annotated on the timeline, including the identification of GLP-1 and GLP-2 through gene sequencing in 1983, the extraction of Exendin-4 from Gila monster venom in 1991, and the first FDA approval of a GLP-1 analog, Exenatide, in 2005. Liraglutide’s clinical trials began in 2000 and the drug’s FDA approval in 2010. Notable is the steep increase in the number of publications from 2000 onwards, reflecting a growing interest and development in the field. The peak in 2022 corresponds to GLP-1 drug sales hitting $22 billion, with the approval of semaglutide by the FDA in 2021 and the initiation of the first clinical trial for an oral diabetes drug based on GLP-1 in the same year. This graph underscores the expanding research and clinical importance of GLP-1-related therapies in the treatment of diabetes. The x-axis represents the year, ranging from 1978 to 2023, and the y-axis quantifies the number of published articles, with a scale ranging from 0 to 2500 articles annual. GLP-1RAs are artificial protein formulations that exhibit partial or complete amino acid sequence similarity to endogenous GLP-1 within the human body. These compounds possess enhanced stability, extended half-lives, and heightened biological potency, enabling them to mimic the actions of GLP-1. Ongoing advancements in research and development have resulted in the production of GLP-1RA with increasingly prolonged half-lives

In 1979, Richard Goodman found that anglerfish are an ideal source for pancreatic mRNA due to their concentrated cells. He extracted mRNA, spliced anglerfish DNA into bacteria, and used radioactive probes to find the somatostatin gene. In 1982, they published a paper in the Proceedings of the National Academy of Sciences (PNAS), revealing that the glucagon precursor gene actually encodes three peptides-glucagon and two new hormones expressed in the intestine.^20^ A year later, a research team led by Graeme Bell from Chiron Corporation published two papers in the journal Nature.^21,22^ They cloned and sequenced the preproglucagon gene, discovering GLP-1 and GLP-2 hormones. The focus shifted to GLP-1, with Svetlana Mojsov identifying its insulin-stimulating fragment, GLP-1(7-37), in 1983. This was inspired by prior GIP (glucose-dependent insulinotropic polypeptide) research, highlighting that besides GIP, other substances in the intestine also stimulate insulin secretion. This finding was later combined with research by Joel Habener and jointly published in JCI.^11^ The team of Jens Juul Holst at the University of Copenhagen in Denmark published a report in FEBS Letters, reaching the same conclusion in January 1987.^23^ In December 1987, Stephen Bloom’s team confirmed in a Lancet paper that GLP-1(7-36) is a human intestinal hormone that stimulates insulin production in the pancreas and lowers blood sugar.^24^ GLP-1, used for treating diabetes, is quickly broken down in the body, requiring high doses that can cause side effects like nausea. This led to the development of new drugs similar to GLP-1 but with a longer-lasting effect. John Eng marked a significant breakthrough; he isolated exendin-4, a peptide from the Gila monster’s venom, which is structurally similar to human GLP-1 but more stable. GLP-1 is degraded in the bloodstream in less than a minute, whereas this peptide, consisting of 39 amino acids, exendin-4, could last for more than two hours. This work was eventually published in the JBC journal in August 1991.^25^ In the early 1990s, despite John Eng’s team identifying a potential diabetes treatment in exendin-4, it initially lacked attention and funding for development. Eng personally secured a patent and partnered with Amylin, leading to the FDA-approved diabetes drug exenatide in 2005, the first GLP-1 analog. This sparked further advancements, including Novo Nordisk’s development of more potent analogs like liraglutide and semaglutide, with the latter achieving over $12 billion in sales by 2022. The journey began in the 1980s with research on Gila monster venom, culminating in the discovery of exendin-4, a stable, effective peptide for treating diabetes, demonstrating the progression from initial discovery to blockbuster diabetes medications. In July 2009, they published a paper in Nature Chemical Biology, reporting for the first time that dual agonists targeting GLP-1R and glucagon receptor (GCGR) had a better weight loss effect.^26^ This marked a significant advancement in obesity treatment research, especially in combining multiple drug targets. Matthias Tschöp and Richard DiMarchi developed the first dual and triple agonist weight loss drugs. Eli Lilly and Company is currently researching a dual agonist called tirzepatide, which has outperformed semaglutide in phase 3 clinical trials. Additionally, their under-development triple agonist, Retatrutide, has shown unprecedented weight loss effects in phase 2 clinical trials.^16^ Matthias Tschöp and his team discovered that dual agonists targeting both GLP-1 and GIP receptors (GIPR) are more effective in treating diabetes than those targeting only GLP-1R. These dual agonists were found to reduce blood sugar and increase insulin secretion in mice, monkeys, and humans.^27^ Subsequently, they investigated a triple agonist capable of simultaneously targeting the GLP-1R, the GCGR, and the GIPR. In December 2014, they published a paper in Nature Medicine, showing that the triple agonist’s weight loss effects in mice exceeded those of the dual agonists.^28^ (Fig. 1).

## GLP-1

GLP-1 is a peptide hormone generated through the enzymatic breakdown of proglucagon. It is synthesized in L-cells located in the intestinal mucosa, α-cells found in the pancreatic islet, and neurons residing in the nucleus of the solitary tract.^29^ GLP-1, an endocrine hormone, is secreted by enteroendocrine L-cells located in the distal jejunum, ileum, and colon in response to nutrient ingestion and neuroendocrine stimulation. It originates from the preproglucagon precursor, which undergoes enzymatic processing within intestinal L-cells, ultimately giving rise to GLP-1(1-37) and GLP-1(7-36) amide or GLP-1(7-37) peptide variants.^30,31^ GLP-1 is an incretin hormone that plays a pivotal role in the meticulous control of human blood glucose levels.^32,33^ Nevertheless, its duration of action is rather ephemeral, lasting a mere 1–2 min within the circulatory system under typical physiological conditions.^34,35^ Subsequently, GLP-1 undergoes enzymatic degradation facilitated by dipeptidyl peptidase IV (DPP-4), leading to the loss of its biological efficacy.^36^

## GLP-1R

GLP-1R, a member of the GPCR family, exhibits specific affinity for GLP-1. It predominantly localizes to the cellular membrane of diverse cell types throughout the human body.^37^ Indeed, GLP-1R is present beyond the confines of the pancreas and extends to a multitude of organs and tissues throughout the body. Its extensive distribution and involvement in diverse physiological processes emphasize the important role of GLP-1R beyond the regulation of glucose metabolism.^38–40^ The phenotype observed after GLP-1R knockout (KO) includes various physiological and metabolic changes. GLP-1R plays a role in the central nervous system (CNS) regulation of appetite. GLP-1R KO can lead to increased appetite, contributing to weight gain.^1^ GLP-1R is involved in regulating both insulin and glucagon secretion, and its knockout can impair pancreatic function, thereby affecting the balance of these hormones and glucose homeostasis.^41,42^ GLP-1R is expressed in the cardiovascular system, and its knockout has implications for cardiovascular function.^43,44^ GLP-1R is present in the CNS, and its absence might contribute to alterations in behavior, mood, or cognitive function.^45,46^

## GLP-1RAs

GLP-1RAs result from intricate structural modifications to GLP-1, enabling them to not only replicate the pharmacological functions of GLP-1 but also impede its hydrolysis by DPP-4, thereby extending the drug’s half-life.^47,48^ These synthetic protein preparations exhibit partial or complete amino acid sequence homology with endogenous GLP-1, rendering them resistant to degradation which resulting in a prolonged half-life and heightened biological activity.^47,48^ By emulating the biological activity of natural GLP-1, GLP-1RAs effectively fulfill the role of GLP-1, effectively lowering blood glucose levels without increasing the risk of hypoglycemia and demonstrating favorable safety profiles.^49^ GLP-1RAs represent a pharmacotherapeutic category of injectable hypoglycemic agents that are sanctioned as adjunctive therapies to diet and exercise for the management of type 2 diabetes mellitus (T2DM) in adult patients and potentially hold promise in addressing other medical conditions.^7,50,51^ As researchers dive deeper into their multifaceted properties, these medications exhibit the potential to offer therapeutic benefits across a spectrum of diseases in human populations.

### The development of GLP-1RAs

The Gila monster, which lives in the deserts of the Americas, can eat up to half its own body weight in one sitting, but its blood sugar remains stable, and its metabolic system runs smoothly despite this large amount of food.^52,53^ In 1992, Dr. John Enn discovered an exopeptide in the saliva of the Gila monster which he named exendin-4. Dr. John found that exenatide is an analog of human GLP-1RA, with 53% homology to human GLP-1RA; exenatide can stimulate human insulin secretion and regulate blood glucose levels in the body. This hormone is not readily degraded by DPP-4 to GLP-1 in the human body and can act for 12 h or longer.^25^

In 2005, exenatide was approved by the U.S. Food and Drug Administration (FDA) for use in treating T2DM;^52^ thus began the competition between various GLP-1RAs. GLP-1RAs are being developed to be more similar to the natural GLP-1 hormone found in the human body. This is achieved by replacing and modifying specific amino acids in human GLP-1.^54,55^ By doing so, the resulting product has a higher similarity or homology to the amino acid sequence of human GLP-1. In addition, efforts are being made to extend the duration of action of GLP-1RAs. Currently, some GLP-1RAs require daily injections, which can be inconvenient for patients. Therefore, researchers are working toward developing formulations that can be administered once a week, providing a longer-lasting effect and reducing the frequency of injections.^56,57^ Furthermore, there is a focus on developing GLP-1RAs that can be taken orally without the need for injections. This oral preparation would offer a more convenient and comfortable option for patients, potentially leading to better treatment compliance.^58,59^ Overall, the future research direction of GLP-1RAs involves making them more similar to those in the human body, extending their duration of action, and creating oral formulations for easier administration.^60,61^ These advancements aim to improve patient experience, compliance, and treatment outcomes.

Exenatide is the world’s first GLP-RA product; it was developed in 1995 and approved for marketing in 2005.^60^ Liraglutide was subsequently approved for marketing in February 2010,^62^ and lixisenatide was approved for marketing in the European Union in 2013 for the treatment of T2DM and in the United States in July 2016.^63^ Albiglutide was approved for marketing in the European Union in March 2014 and in the United States in April 2014.^64^ Dulaglutide was approved for marketing in the U.S. in September 2014 for the treatment of T2DM^65^; semaglutide was approved for marketing in the United States in September 2017^66^; and beinaglutide is a GLP-RA product developed by a Chinese company and was approved for marketing for the treatment of T2DM in China in September 2016.^67^ PEG-loxenatide, China’s first long-acting GLP-RA product, was approved for marketing in China in May 2019.^66^ (Tables 1 and 2)

**Table 1:** Comparison of GLP-1RAs used in type 2 diabetes mellitus

Date	Brand name	GLP-1RA	Half-life	Dose (SC)	Molecular formula	Clinial trial focus
2005	Byetta	Exenatide	2.4 h	5 μg or 10 μg b.i.d sc	C_149_H_234_N_40_O_47_S	Parkinson disease
2010	Victoza/Saxenda	Liraglutide	11-13 h	0.6 mg and 1.2 or 1.8 mg q.d sc	C_172_H_265_N_43_O_51_	Cardiovascular disease
2013	Adlyxin (U.S)/Lyxumia (EU)	Lixisenatide	2–4 h	10 μg then 20 μg q.d sc	C_215_H_347_N_61_O_65_S	Early Parkinson disease
2014	Tanzeum	Albiglutide	6-8 h	30 or 50 mg q.w sc	C_148_H_224_N_40_O_45_	Cardiovascular disease
2014	Trulicity	Dulaglutide	108-112 h	0.75 or 1.5 mg q.w sc	C_2646_H_4044_N_704_O_836_S_18_	Type 2 diabetes
2017	BYDUREON BCise	Exenatide (ER)	2 w	2 mg q.w sc	C_184_H_282_N_50_O_60_S	Type 1 diabetes
2017	Ozempic	Semaglutide	6-7 d	0.25 mg then 0.5 mg q.w sc	C_187_H_291_N_45_O_59_	obesity-related heart failure
2019	Rybelsus	Oral Semaglutide	24 h	3 mg then 7 mg q.d po	C_187_H_291_N_45_O_59_	Type 2 diabetes

Form: Pen, SC injection; po, orally

*q.d* once daily, *q.w* once weekly, *b.i.d* twice daily, *t.i.d* three times daily, *SC* subcutaneous administration, *w* week, *po* orally

Date: Refers to the date when the drug was approved by the U.S. Food and Drug Administration (FDA)

Clinical Trial Focus: Describes the main diseases targeted in recent clinical trials

**Table 2:** Comparative overview of efficacy and adverse effects of mainstream GLP-1RA medications

	CV benefit	Renal benefit	Weight loss	Patient adherence	HbA1c reduction	HbA1c problem	Injection reactions	GI side effects	Antibody issues
Exenatide					+	−−	−−	−	−−
Lixisenatide					+	−−	−−	−	−
Oral Semaglutide	++				+	−−		−−	
Dulaglutide	++	+		++	++	−−−			
Liraglutide		+	++	++	++	−−		−−	
Exenatide (ER)				++	++	−			−
Semaglutide (S.C)	+++	+++	+++	++	++			−	

*CV* cardiovascular, *GI* gastrointestinal, *RE* extended-release, *SC* subcutaneous administration

The “+“ and “−“ symbols indicate the efficacy profile or adverse effect profile of each parameter, with more symbols meaning greater strength or severity. More “+“ signs indicate a greater benefit. More “−“ signs indicate more severe side effect issues

### Current GLP-1RAs

GLP-1RAs are synthetic protein preparations that have partial or complete amino acid sequence homology with GLP-1 found in the body, are not easily degraded, have longer half-lives and have stronger biological activity. They can play the role of GLP-1.^49^ GLP-1RA is primarily used for the treatment of T2DM^68^ and works by mimicking the action of the naturally occurring hormone GLP-1, which is released by the intestines in response to food intake. GLP-1 helps regulate blood sugar levels by stimulating the release of insulin, suppressing glucagon secretion (which raises blood sugar levels), and slowing the rate at which the stomach empties, leading to a feeling of fullness and reduced appetite.^69^ In addition to its use in diabetes management, GLP-1RA has also shown potential in other areas of research. Studies have explored its effects on weight loss, NAFLD,^70^ and neurodegenerative diseases such as AD.^71^ Furthermore, GLP-1RA has been investigated for its potential to reduce the risk of cardiovascular events in patients with or without diabetes.^5,19^ Like any medication, GLP-1RAs may have side effects, including nausea, vomiting, diarrhea, and injection site reactions. These side effects are often mild and tend to improve over time.^72–74^ Rare but serious side effects may include pancreatitis and allergic reactions.^75^

#### Exenatide

Exenatide is a medication that belongs to the class of drugs known as GLP-1RAs. It is a synthetic version of the hormone exendin-4, which is found in the saliva of the Gila monster, a venomous lizard native to the southwestern United States.^76^ Exenatide is composed of 39 amino acids and shares 53% sequence identity to human GLP-1. The second position of its N-terminus is glycine (alanine in GLP-1), which is not easily degraded by the DPP-4 enzyme.^77^ Compared with GLP-1, the C-terminus of its amino acid sequence contains 9 additional amino acid residues (PSSGAPPPS), which are not easily degraded by peptide chain endonucleases; thus, it has a long half-life and strong biological activity.^78^ The average half-life of exenatide is 2.4 h, 2 to 3 times per day.^78^

#### Liraglutide

Liraglutide is a medication used to treat T2DM and obesity.^79,80^ In the treatment of T2DM, liraglutide helps lower blood sugar levels by increasing insulin production and reducing glucose production by the liver.^81^ It also slows stomach emptying, which helps control appetite and reduce food intake.^82,83^ In addition to lowering blood sugar levels, liraglutide helps to reduce body weight by suppressing appetite and reducing energy intake,^84,85^ but its associated costs and need for daily injections may limit its use in individuals with obesity.^86–88^ Liraglutide was first approved by the FDA in 2010 under the brand name Victoza for the treatment of T2DM.^83,89–92^ Since then, it has also been approved for other indications, such as chronic weight management, under the brand name Saxenda.^62^ Liraglutide has an arginine at position 34 (GLP-1(7-37)) that is lysine, and its lysine at position 26 connects to a 16-carbon palmitic acid side chain linked by glutamic acid. Liraglutide shares 97% sequence homology with human GLP-1 and can bind to and activate GLP-1R.^78^ The elimination half-life of liraglutide is 13 h, and only one injection per day is required.^62^

#### Lixisenatide

Lixisenatide was developed by Sanofi to treat T2DM.^64,93^ Structurally, lixisenatide is based on the exenatide structure but lacks proline at position 38, and six lysines are linked after serine at position 39.^94^ The six lysine residues increase the rigidity of the molecule’s structure, thus allowing its drug properties to be improved.^78^ These changes stabilize its structure, prevent protein degradation of the molecule in the circulation, and increase the circulation time enough to ensure once-daily injection (compared with exenatide, which is injected two or three times daily). The average half-life of lixisenatide is approximately 3–4 h.^95^ Lixisenatide is a short-acting GLP-1RA agent,^96,97^ and once-daily lixisenatide can improve patient compliance to a certain extent and reduce the occurrence of hypoglycemia.^98–100^ The injection is usually given within one hour before the first meal of the day, preferably at the same time each day. It is important to avoid injecting lixisenatide after a meal or in case of a missed meal. The initial recommended dosage of lixisenatide is 10 mcg once daily for at least 14 days. After the initial period, its dosage may be increased to 20 mcg once daily if additional glycemic control is needed. The maximum recommended dosage is 20 mcg once daily.^101^

#### Albiglutide

Albiglutide was approved by the FDA in 2014. As a GLP-1RA,^102^ albiglutide works by stimulating insulin release and reducing glucagon production, leading to improved blood sugar control in patients with T2DM.^102,103^ Albiglutide is a long-acting GLP-1RA injected subcutaneously once a week. The half-life of albiglutide is approximately 4 to 7 days.^102^ Compared with the structure of human GLP-1(7-36), the amino acid sequence of albiglutide contains an arginine at position 8 (in GLP-1, this residue is lysine), and two modified GLP-1 peptide chains are fused to human serum albumin (HSA), thereby greatly extending its half-life.^104,105^ Albiglutide is typically prescribed in combination with diet and exercise. The dosage of albiglutide is usually 30 mg once a week. It is administered as a subcutaneous injection (just under the skin). The injection site can be the thigh, abdomen, or upper arm.^102^

The FDA set several usage restrictions upon the initial approval of Tanzeum (albiglutide), reflecting considerations of suitability and safety for specific patient groups. Firstly, Tanzeum is not recommended as a first-line treatment for patients inadequately controlled with diet and exercise.^49^ Secondly, its safety and efficacy remain unclear in patients with a history of pancreatitis as it has not been studied in this group.^106^ Additionally, Tanzeum is not suitable for treating type 1 diabetes or diabetic ketoacidosis, related to its pharmacological action and target disease. It is also not recommended for patients with existing gastrointestinal disease to avoid potential side effects or exacerbating the condition.^107^ Further, serious risks associated with Tanzeum use include pancreatitis, acute kidney injury, renal impairment, and pneumonia, further limiting its use in specific conditions or susceptible patients. These restrictions and warnings demonstrate the FDA’s stringent consideration of patient safety in the drug approval process.^108^ In March 2015, the FDA required a black box warning on Tanzeum due to the observed risk of thyroid C-cell tumors in animals, although it is unclear if this effect also occurs in humans.^108^ Later, the FDA added a warning about the risk of anaphylactic shock to the medication’s label. This reaction is severe and potentially life-threatening, including symptoms like unease, tingling, dizziness, itching, hives, swelling, difficulty breathing, and fainting.^81,107,109^

Tanzeum was discontinued by GlaxoSmithKline (GSK) in 2017, primarily due to economic factors.^110,111^ Despite GSK’s attempts to gain a competitive edge through low pricing, Tanzeum failed to achieve sufficient market acceptance.^106^ In 2017, Tanzeum was removed from the preferred drug list of leading pharmacy benefit manager (PBM) company Express Scripts and was replaced by Eli Lilly’s Trulicity, highlighting Tanzeum’s weak market presence.^106,108^ Moreover, this decision was part of a broad strategic reform led by GSK’s new CEO Emma Walmsley.^106^ This reform aimed to refocus the company’s efforts on areas with higher revenue potential, such as respiratory and HIV treatments, as well as oncology and immunology.^108^ Additionally, Tanzeum struggled to establish a significant market share in the competitive GLP-1RA market, which was another reason GSK decided to withdraw the drug globally.^108^

#### Dulaglutide

Dulaglutide was approved by the FDA in 2014 for the treatment of T2DM.^112^ Dulaglutide is a once-weekly long-acting GLP-1RA. Compared with the structure of human GLP-1(7-37), the amino acid sequence of dulaglutide contains glycine at position 8, glutamic acid at position 22, and glycine at position 36.^113^ It was then fused to the constant region (Fc) of modified human immunoglobulin G4 (IgG4) via a “-(Gly-Gly-Gly-Gly-Ser) 3-Ala-“ bridge and exhibits an average biological half-life of 90 h.^105^ Dulaglutide is the first large-molecule GLP-1RA, and the weekly dosing frequency of dulaglutide can greatly improve patient compliance.^33,78,104,114^

#### Semaglutide

Semaglutide is a long-acting GLP-1RA agent used once weekly to improve glycemic control in patients with T2DM.^115^ Compared with the structure of human GLP-1(7-37), the amino acid sequence of semaglutide contains diaminoisobutyric acid at position 8, arginine at position 34, and acylated lysine at position 26.^31^ Semaglutide has a longer aliphatic chain and increased hydrophobicity, but its hydrophilicity is greatly enhanced by PEG modification of the short chain. Modified DPP-4 can not only mask the enzymatic hydrolysis site of DPP-4 but also bind closely with albumin to reduce renal excretion and prolong the half-life.^35,104,116^ On August 22, 2022, Novo Nordisk announced the primary results of the Phase II clinical trial for the dual-action compound CagriSema, which demonstrated effective blood sugar reduction and weight loss. CagriSema is composed of the GLP-1 RA semaglutide and the long-acting insulin analog cagrilintide, and can be administered subcutaneously once a week.^117^

#### Beinaglutide

Beinaglutide was the first original drug approved for the treatment of obesity in China and the third GLP-1 class reduction drug approved worldwide, and it represents a new treatment option for overweight and obese patients.^67,118,119^ Beinaglutide, a recombinant human GLP-1 (rhGLP-1) polypeptide, exhibits a remarkable resemblance to human GLP-1(7-36), with nearly 100% homology.^120^ This innovative compound demonstrates dose-dependent efficacy in regulating glycemic control, suppressing appetite, delaying gastric emptying, and facilitating weight reduction.^121^ Consequently, beinaglutide holds significant promise for advancing research in the areas of overweight/obesity and nonalcoholic steatohepatitis (NASH).^67,122^ Beinaglutide is a natural human GLP-1(7-36)-NH2 expressed in Escherichia coli. This product was approved for the treatment of T2DM; it has a half-life of 11 minutes and requires 3 injections per day.^78^

#### Polyethylene glycol liraglutide

Polyethylene glycol liraglutide (PEG-loxenatide) is a long-acting GLP-1RA. It was the world’s first PEGylated long-acting GLP-1RA.^123^ PEG-loxenatide is used for blood glucose control in adult patients with T2DM. Structurally, the product was optimized on the basis of exenatide, and the glycine 2, methionine 14 and asparagine 28 positions were modified to improve enzyme stability and chemical stability based on the polypeptide backbone. Moreover, the C-terminal serine of the peptide was replaced by cysteine via site-directed mutagenesis performed with polyethylene glycol.^78^ The half-life of PEG-loxenatide is approximately 1 week.^123^

#### Multi agonists

In recent years, the development of dual and triple agonists related to GLP-1 has been vigorously pursued.^124–126^ The design concept of these multi-agonists is to simultaneously regulate multiple key metabolic pathways to achieve more effective control over blood sugar, body weight, and overall metabolic health.^43^ Currently, these drugs are primarily in the development and clinical trial stages, but preliminary research results have shown their potential powerful effects in reducing blood sugar and body weight.^127,128^

##### Dual agonists

Dual agonists target both the GLP-1R and another specific receptor.^124,129^ A common combination is GLP-1 with GIP or insulin-like growth factor.^130^ For example, a popular dual agonist, tirzepatide (brand name Mounjaro), activates both GLP-1 and GIPR.^124^ GLP-1, by activating its receptor, increases insulin secretion and reduces glucagon secretion, thereby lowering blood sugar levels.^131–133^ Additionally, GLP-1 helps to delay gastric emptying and suppress appetite, aiding in weight management.^121,134^ GIP, another insulin secretion agonist, helps release insulin, particularly after eating, enhancing the effects of GLP-1 and thereby improving the overall therapeutic efficacy of the drug.^125,130^ In 2022, tirzepatide was approved by the FDA for the treatment of T2DM in the United States.^135,136^ It is considered a significant breakthrough in diabetes treatment and is also being studied for the treatment of obesity due to its significant weight loss effects.^135^ Tirzepatide is developed by Eli Lilly and has proven to provide superior blood sugar control and significant weight loss, making it particularly valuable in the treatment of T2DM.^136–138^ Research and clinical trials have shown that tirzepatide not only improves blood sugar levels but also has a positive impact on cardiovascular risk factors.^139–141^ Although dual agonists show advantages in efficacy, their safety and tolerability continue to be a focus of ongoing monitoring.^141^ Common side effects include gastrointestinal reactions, such as nausea and vomiting, which are typically more common during the initial stages of treatment.^141,142^ The Phase III clinical trials of tirzepatide were conducted in 77 research centers across seven countries, including the United States, Brazil, and Japan.^143^ The trials recruited adult participants with T2DM and significantly reduced body weight and improved blood sugar control in these patients.^143^ The safety profile of tirzepatide is similar to other drugs in its class, providing an effective new treatment option for patients with T2DM and obesity. Current dual agonists under investigation also include efinopegdutide and cotadutide.^144–147^

##### Triple agonist

Triple agonists go even further.^148^ These drugs act by simultaneously targeting three different agonists GLP-1R, the GIPR, and the GCGR.^149–151^ These receptors each have independent yet complementary roles in the treatment of diabetes and obesity.^150,152,153^ Activation of the GLP-1R can enhance insulin secretion, reduce glucagon secretion, delay gastric emptying,^154^ and suppress appetite.^155,156^ GIPR activation also promotes insulin release, especially after meals, helping to improve glucose utilization.^157,158^ Activation of the insulin or Insulin-like Growth Factor 1 (IGF-1) receptor can enhance insulin sensitivity, improve glucose absorption and utilization by cells, and potentially have positive effects on cardiovascular health and long-term energy balance.^159^ As of now, GLP-1-related triple agonists are primarily still in the development stage and have not been widely approved for use.^159^ These drugs are not yet widely available on the market but have shown some potential in clinical trials.^160,161^ For example, HM15211, a triple agonist developed by Hanmi Pharmaceutical in South Korea that activates GLP-1R, GIPR and GCGR has entered early clinical trials for the treatment of obesity and non-alcoholic steatohepatitis (NASH).^162,163^ Retatrutide (LY-3437943), a novel triple agonist developed by Eli Lilly that targets GLP-1R, GIPR and GCGR, has shown potential in preliminary clinical data for providing excellent blood sugar control and significant weight reduction.^158,161,164,165^ However, activating multiple receptors may lead to more complex side effects, and in some experiments, dual and triple agonists have indeed shown more severe side effects.^165,166^ Retatrutide has now entered Phase III clinical trials.^161^ The results of the Phase II clinical trials of retatrutide were published in The Lancet in 2023.^161^ The trials involved adult participants with T2DM aged 18 to 75, conducted across 42 research and medical centers in the United States.^161^ Over a period of 24 weeks, all dosage groups of retatrutide showed significant improvements in reducing glycated hemoglobin (HbA1c) and body weight compared to the placebo group, especially in the higher dosage groups. In terms of safety and tolerability, the adverse events were primarily mild to moderate gastrointestinal reactions, with no reports of severe hypoglycemia or death.^161^

In summary, dual and triple agonists related to GLP-1 represent significant advances in the field of diabetes treatment,^167,168^ demonstrating the future trend of enhancing therapeutic effects by targeting multiple biomarkers.^136,169,170^ With the accumulation of more clinical data and the development of new drugs, these treatment options are expected to provide more effective and comprehensive treatment choices for diabetes patients.^161,167^

#### Small molecule GLP-1RAs

Currently, most GLP-1RAs are based on proteins or peptides, meaning they are large molecules typically administered via injection.^37^ Small molecule GLP-1RAs are chemically synthesized, and compared to protein-based drugs, they generally have smaller molecular sizes.^171^ The development of small molecule GLP-1RAs aims to overcome some of the limitations of traditional protein or peptide-based GLP-1RAs, such as the need for injection. Small molecule drugs may offer the possibility of oral administration, which is more convenient and acceptable for patients.^171^ Additionally, small molecule drugs may have better tissue permeability, longer half-lives in the body, and lower production costs. As of now, research on small molecule GLP-1RAs is still mainly in the laboratory and early clinical trial stages.^172^ The challenges in developing these drugs include ensuring that they can effectively mimic the biological activity of large molecule GLP-1RAs while maintaining efficacy and selectivity. This review article will introduce some of the small molecule drugs that are currently receiving significant attention:

##### Orforglipron

Orforglipron is an oral small molecule GLP-1RA developed jointly by Eli Lilly and Chia Tai Tianqing Pharmaceutical Group.^173^ Its research findings were recently presented orally at the 83rd Scientific Sessions of the American Diabetes Association and published in the New England Journal of Medicine. In a 26-week study, orforglipron demonstrated a significant dose-dependent effect on weight loss, with weight reduction ranging from 8.6% to 12.6% across various dosages, compared to only 2.0% in the placebo group. By week 36, this weight loss effect was even more pronounced, increasing from 9.4% to 14.7%, while the placebo group saw a reduction of only 2.3%. Additionally, in another Phase II study targeting patients with T2DM, orforglipron also showed significant effects in reducing A1C and weight, achieving the study’s primary and secondary endpoints. In this study, participants taking orforglipron experienced an average A1C reduction of 2.1% and an average weight loss of 10.1 kilograms at 26 weeks, which was significantly greater than those in the placebo and dulaglutide groups. Between 65% and 96% of participants taking orforglipron achieved an A1C level below 7.0% at 26 weeks.^174^ Currently, Eli Lilly has initiated a Phase III development program to further investigate the efficacy and safety of orforglipron in treating obesity, overweight, and T2DM.

##### Danuglipron

Danuglipron is an oral GLP-1RA developed by Pfizer. In May 2023, Pfizer released the results of a Phase 2b clinical trial of the drug. The study involved 411 adult patients with T2DM and was designed as a randomized, double-blind, placebo-controlled trial where patients received varying doses of danuglipron or a placebo. The results showed that during the 16-week treatment period, patients who received the highest dosage (120 mg twice daily) of danuglipron experienced an average reduction in HbA1c of 1.16 percentage points and a weight loss of 4.17 kilograms. All dosages of danuglipron significantly reduced patients’ HbA1c and fasting blood glucose levels, with more pronounced weight loss effects observed in the 80 mg and 120 mg doses compared to the placebo group. Common adverse reactions included nausea, diarrhea, and vomiting.^172^

##### GSBR-1290

GSBR-1290 is an oral small molecule GLP-1RA developed by Structure Therapeutics, aimed at treating T2DM and obesity. On December 18, 2023, Structure Therapeutics published the latest clinical data for GSBR-1290 on its official website.^175^ Currently, GSBR-1290 is undergoing a 12-week Phase 2a randomized, double-blind, placebo-controlled clinical trial to assess its effectiveness in treating patients with T2DM and obesity. To date, the trial has enrolled 94 participants, with 54 in the T2DM group and 40 in the obesity group. Regarding safety, the majority of reported adverse events were mild to moderate, ranging from 88% to 96%, depending on the specific study group. Among the 60 participants treated with GSBR-1290, only one (2.8%, from the T2DM group) discontinued the study due to drug-related adverse events (AEs). As for clinical outcomes, in the T2DM group, there was a significant reduction in HbA1c (decreased by 1.01% to 1.02%, placebo-adjusted) and a clinically meaningful decrease in body weight of 3.26% to 3.51% after 12 weeks of treatment. In the obesity group, there was a significant and clinically meaningful reduction in body weight of 4.74% at week 8, with weight continuously decreasing during the 8-week treatment period.^175^

## Classical pathophysiological mechanisms of GLP-1

### GLP-1 signaling pathway

GLP-1 initiates signaling by binding to its receptor, GLP-1R, which is a G-protein-coupled receptor.^176,177^ When GLP-1 binds to GLP-1R, it triggers the activation of G-proteins, leading to an increase in the intracellular second messenger cAMP.^34,39,177^ The rise in cAMP activates protein kinase A (PKA), which then promotes the synthesis and secretion of insulin and inhibits the release of glucagon.^178,179^ Additionally, cAMP can activate Rap1 through EPAC (Exchange Protein directly Activated by cAMP),^180–182^ which is involved in regulating insulin secretion.^180,181,183^ GLP-1 also activates the phosphoinositide 3-kinase (PI3K)/protein kinase B (Akt) pathway, which is crucial for maintaining the survival and function of pancreatic β-cells.^184–187^

### Interactions with other pathways

GLP-1 not only promotes the release of insulin but also enhances the response of pancreatic β-cells to insulin through the PI3K/Akt pathway, thereby improving insulin signal transduction and increasing the sensitivity of peripheral tissues to insulin.^1,188^ GLP-1 reduces hepatic glucose production, partly by inhibiting the expression and activity of key gluconeogenic enzymes.^189–191^ Activation of GLP-1R leads to the production of cAMP (cyclic Adenosine Monophosphate), which is achieved by activating Adenylyl Cyclase (AC).^191,192^ Following the activation of GLP-1R, the βγ subunits of GPCRs can directly activate Class I PI3Ks.^1^ These PI3Ks typically include the PI3Kα and PI3Kβ isoforms, which are composed of regulatory subunits containing SH2 domains and catalytic subunits.^193^ These subunits can directly interact with the activated GPCR or do so via intermediary proteins such as insulin receptor substrate.^194,195^ The activated PI3K catalyzes the conversion of membrane phospholipid Phosphatidylinositol-4,5-bisphosphate (PIP2) into Phosphatidylinositol-3,4,5-trisphosphate (PIP3).^196^ The generation of PIP3 is a crucial step for the activation of Akt, as PIP3 provides a membrane docking site for Akt, facilitating its translocation to the cell membrane.^197^ Akt, also known as Protein Kinase B (PKB), once positioned at the membrane, can be phosphorylated by PIP3-dependent kinase 1 (PDK1) and possibly PDK2 (such as mTORC2).^196–198^ The phosphorylation of Akt is necessary for its full activation, allowing it to regulate a variety of downstream effector proteins involved in cell survival, proliferation, metabolism, and glucose transport.^199^ Activated Akt promotes the expression and translocation of GLUT4 (Glucose Transporter Type 4) to the cell membrane, increasing cellular glucose uptake.^200–202^ Simultaneously, Akt promotes cell survival by phosphorylating and inhibiting a series of pro-apoptotic proteins, such as Bad and the FOXO family.^202–204^ Moreover, Akt can activate mTORC1, further promoting cell growth and protein synthesis. Through these molecular mechanisms, GLP-1 not only plays a crucial role in the treatment of diabetes by enhancing the function and protecting pancreatic β-cells from apoptosis, but it may also offer potential therapeutic benefits in fields such as cardiovascular and neural protection.^198,205,206^ GLP-1 can also enhance the uptake and utilization of glucose in muscle and adipose tissues.^207–209^ And GLP-1’s action in the brain reduces appetite and may influence energy expenditure.^176,210,211^ These effects involve interactions with other satiety and hunger signals, such as PYY, CCK, insulin, and leptin.^212,213^

### The pathophysiological mechanism of GLP-1 in metabolic diseases

GLP-1 plays a crucial role in the pathophysiology of metabolic diseases, particularly in T2DM and obesity.^213–215^

#### Role of GLP-1 in T2DM

GLP-1 significantly influences the functionality of pancreatic β-cells and α-cells, contributing to its therapeutic effect on T2DM.^132,216,217^ As a vital incretin hormone, GLP-1 enhances glucose-dependent insulin secretion.^3^ It also promotes proliferation and reduces apoptosis of pancreatic β-cells, thereby maintaining their quality and functionality.^130,218^ At the molecular level, GLP-1 activates the cAMP response element-binding protein (CREB) via its receptor (GLP-1R), a transcription factor crucial for expressing the insulin gene.^3,130,219^ GLP-1 also activates PKA and EPAC through a cAMP-dependent pathway.^1,220^ PKA, a key enzyme, phosphorylates various target proteins, affecting their activity and function, which in turn promotes insulin synthesis and secretion.^178,180^ The PI3K/Akt signaling pathway, activated by GLP-1, plays a vital role in maintaining pancreatic β-cell survival and promoting their proliferation.^221,222^ Activation of Akt stimulates β-cell proliferation, reduces apoptosis, and enhances insulin secretion by regulating downstream effector molecules like Forkhead box protein O1 (FoxO1) and the glucose transporter type 2 (GLUT2).^223,224^

GLP-1 also inhibits glucagon release from α-cells, which is beneficial for reducing blood glucose levels since glucagon promotes hepatic gluconeogenesis.^131,225^ GLP-1’s action on α-cells regulates glucagon release.^131^ The direct impact of GLP-1 on these cells slows the secretion of glucagon, essential for maintaining glucose stability, especially postprandially.^131,226^ When GLP-1 binds to its receptor on α-cells, it activates intracellular cAMP production.^227^ In α-cells, increased cAMP affects glucagon synthesis and release.^228^ PKA, activated by cAMP, can regulate the activity of K-ATP channels in α-cells.^228,229^ The opening of these channels is controlled by the intracellular ATP/ADP ratio.^229^ Specifically, PKA modifies the open state of K-ATP channels through phosphorylation, affecting the cell membrane’s potential and intracellular calcium ion concentration.^230,231^ By modulating the activity of K-ATP channels, GLP-1 indirectly controls the calcium signaling in α-cells, thereby influencing glucagon secretion.^232,233^ GLP-1 inhibits the release of glucagon from α-cells through multiple mechanisms. GLP-1 can directly bind to the receptors on the surface of α-cells in the pancreas, inhibiting the secretion of glucagon from these cells.^234,235^ Glucagon is a hormone secreted by pancreatic α-cells that stimulates the liver to release glucose. Therefore, inhibiting the release of glucagon is crucial when blood glucose levels need to be lowered. GLP-1 can stimulate pancreatic β-cells to release insulin.^236,237^ Insulin not only directly lowers blood glucose levels but also further inhibits glucagon secretion from α-cells through a feedback mechanism.^238^ The high concentration of insulin in the local pancreatic environment creates a negative feedback effect, reducing the amount of glucagon secreted by α-cells.^234^ Additionally, GLP-1 can reduce blood glucose production by delaying gastric emptying and decreasing appetite, which also contributes to overall blood glucose control.^239,240^ In summary, GLP-1 lowers blood glucose levels and inhibits glucagon secretion through direct action on α-cells, promotion of insulin secretion, and regulation of gastrointestinal activity.^241^

GLP-1R is expressed not only on β-cells and α-cells but also on δ-cells in the pancreatic islets.^242^ δ-cells primarily secrete somatostatin, which is an important regulatory hormone.^242^ The expression of GLP-1R on δ-cells helps to inhibit the secretion of glucagon.^242^ When GLP-1 binds to GLP-1R on δ-cells, it activates these cells and promotes the secretion of somatostatin.^243^ Somatostatin is a potent inhibitory hormone that affects the surrounding α-cells and β-cells. It acts directly on neighboring α-cells to inhibit the secretion of glucagon and can also indirectly influence α-cells by inhibiting the secretion of other hormones.^243^ In the local islet environment, a high concentration of somatostatin creates an inhibitory milieu, further reducing the activity of α-cells and the secretion of glucagon.^244^ Additionally, somatostatin can inhibit gastrointestinal activity, reducing the secretion of pancreatic enzymes and gastric acid, thereby indirectly decreasing the demand for glucagon.^245,246^ Hence, GLP-1 activates δ-cells and promotes somatostatin secretion, forming a multi-layered inhibitory mechanism that effectively reduces glucagon secretion.

The relative expression levels of GLP-1R on α-cells and δ-cells exhibit certain differences. These variations significantly influence the role of GLP-1 in regulating islet function and glucose homeostasis.^131^ Research indicates that the expression level of GLP-1R on α-cells is relatively low.^131^ Although GLP-1Rs are present, they are limited in number, making the direct inhibitory effect of GLP-1 on α-cells relatively weak.^247^ The primary action often occurs through indirect mechanisms, such as insulin and somatostatin.^244^ Conversely, the expression level of GLP-1R on δ-cells is relatively high.^248^ GLP-1 can effectively bind to receptors on δ-cells, stimulating the secretion of somatostatin.^248^ As a broad-spectrum inhibitory hormone, somatostatin can effectively inhibit glucagon secretion from α-cells and insulin secretion from β-cells.^249,250^ Relevant studies suggest that the expression of GLP-1R on δ-cells is crucial for GLP-1’s regulation of somatostatin secretion and the overall inhibitory effect on glucagon.^248,251,252^ This mechanism is particularly evident in the use of GLP-1-based drugs, such as GLP-1RAs, in the treatment of T2DM. In summary, the relatively higher expression of GLP-1R on δ-cells allows GLP-1 to indirectly inhibit glucagon secretion by promoting somatostatin secretion. In contrast, the lower expression of GLP-1R on α-cells results in a limited direct effect. Although these three types of cells each have distinct functions, it is more important to note that the somatostatin, glucagon, and insulin they secrete work together through mutual regulation and feedback mechanisms to maintain blood glucose balance and overall metabolic homeostasis.^253,254^

**Fig. 2:**
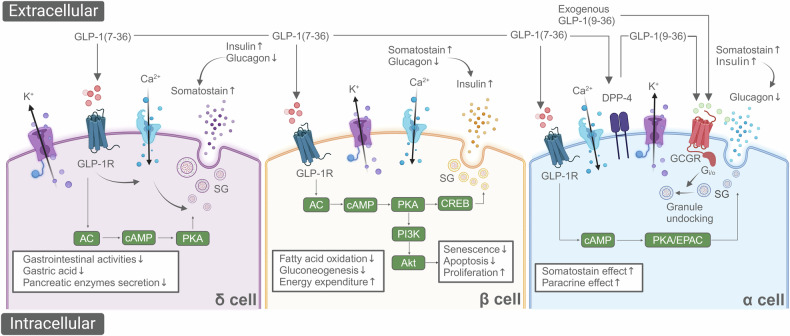
Mechanisms of Blood Glucose Reduction by GLP-1 in Pancreatic α, β, and δ Cells. This illustration demonstrates how GLP-1 reduces blood glucose levels by acting on different pancreatic cell types. In β cells, GLP-1(9–36) activates the GLP-1R, which increases cAMP levels, subsequently activating PKA and CREB, leading to the promotion of insulin secretion. The PI3K/Akt pathway enhances glucose sensitivity in β cells, promoting insulin secretion. This pathway also supports β cell survival and proliferation, ensuring an adequate β cell mass to maintain normal insulin secretion. This signaling cascade results in increased insulin gene expression, enhanced protein synthesis, improved cell survival, and reduced apoptosis. Additional metabolic effects include fatty acid oxidation, gluconeogenesis, and energy expenditure. In α cells, GLP-1(7-36) mainly regulates function through indirect mechanisms. GLP-1R expression is lower in α cells compared to β and δ cells, resulting in relatively less direct action of GLP-1 on α cells. Through paracrine effects via β cells, GLP-1 enhances insulin secretion, which in turn inhibits glucagon secretion from α cells. Additionally, GLP-1 promotes somatostatin secretion from δ cells, which inhibits glucagon secretion from α cells. GLP-1(7-36) can suppress glucagon secretion in α cells by increasing cAMP levels, activating PKA and EPAC, leading to a decrease in intracellular calcium concentration and reduced glucagon release. GLP-1(9–36) inhibits glucagon secretion by activating inhibitory G proteins (Gi/o) and suppressing PKA activity through a GCGR-dependent mechanism. By promoting the undocking of secretory granules (SG), GLP-1(9–36) reduces the number of granules available for exocytosis, thereby decreasing the release of glucagon. δ cells primarily secrete somatostatin. GLP-1(7-36) regulates somatostatin secretion by modulating calcium channels and affecting membrane potential changes. The action of GLP-1(7-36) on δ cells may be more indirect, such as through the influence on hormones secreted by β and α cells (insulin and glucagon), which indirectly affects δ cell somatostatin secretion. Additionally, somatostatin can inhibit gastrointestinal activities, reducing the secretion of pancreatic enzymes and gastric acid, thereby indirectly lowering the demand for glucagon

GLP-1 usually refers to GLP-1(7-36), a peptide chain consisting of 36 amino acids and the primary active molecule.^255^ GLP-1(7-36) stimulates the release of paracrine glucagon inhibitory factors by activating GLP-1R on β-cells and δ-cells.^241^ DPP-4 cleaves GLP-1(7-36) at the 8th position, generating GLP-1(9–36). GLP-1(9–36) is the product of GLP-1(7-36) degradation by DPP-4, and this process rapidly reduces the activity of GLP-1(7-36).^35,256,257^ Traditionally, GLP-1(9–36) was considered a metabolically inactive product of GLP-1, losing its primary function of regulating blood sugar.^258^ However, increasing research suggests that GLP-1(9–36) may have other physiological roles. Recent studies show that GLP-1(9–36) can activate the inhibitory G protein (Gi/o), leading to the translocation of secretory granules (SG) beneath the cell membrane, thereby inhibiting glucagon secretion.^241^ This mechanism is unaffected by genetic or pharmaceutical inhibition of GLP-1R, but it is sensitive to pertussis toxin. As exendin-4 is more resistant to DPP-4-induced degradation, this mechanism is not activated by exendin-4.^241^ GLP-1(9–36) can directly act on pancreatic α-cells to inhibit the secretion of glucagon. Recent studies have shown that GLP-1(9–36) is particularly effective at low glucose concentrations, with its inhibitory effect being similar to that of GLP-1(7-36).^241^ GLP-1(9–36) retains its ability to inhibit glucagon secretion even after GLP-1R inactivation, suggesting that its mechanism of action may not be entirely dependent on the GLP-1R.^241^ GLP-1(9–36) promotes the undocking of secretory granules (SG) by inhibiting the entry of Ca^2+^ through voltage-gated Ca^2+^ channels. As a result, GLP-1(9–36) reduces the number of granules available for exocytosis in α-cells, thereby decreasing the release of glucagon.^241^ This mechanism decreases intracellular Ca^2+^ concentration, thereby inhibiting glucagon secretion.^241^ Additionally, GLP-1(9–36) can effectively inhibit glucagon secretion induced by β-adrenergic stimulation, amino acids, and membrane depolarization, indicating its inhibitory effect under various stimulatory conditions.^241^ In α-cells of patients with T2DM, the ability of GLP-1(9–36) to inhibit glucagon secretion is lost. This may be due to altered α-cell function in diabetic patients, which impairs the efficacy of GLP-1(9–36).^241^ In vivo experiments have shown that high concentrations of exogenous GLP-1(9–36) can lower circulating glucagon levels during insulin-induced hypoglycemia. However, this effect is significantly diminished or absent in diabetes.^241^ As a degradation product of GLP-1(7-36), GLP-1(9–36) has a notable inhibitory effect on glucagon secretion, demonstrated in both in vitro and in vivo studies. However, this inhibitory effect is significantly weakened in patients with T2DM, indicating that its potential application in diabetes treatment requires further investigation.^241^ Through this series of complex molecular and cellular mechanisms, GLP-1 plays a vital role in the physiological and pathological processes of metabolic diseases like T2DM.^232,233,241,259^ (Fig. 2).

#### Role of GLP-1 in obesity

GLP-1 reduces appetite and food intake through its actions in the CNS.^212,260,261^ By interacting with receptors in the hypothalamus, GLP-1 influences satiety and reduces the consumption of food in both animals and humans.^262^ It functions not only in peripheral tissues but also directly within the CNS, crossing the blood-brain barrier or being produced centrally to act on the brain.^262,263^ GLP-1 regulates energy balance and food intake by acting on specific brain regions, especially the hypothalamus, a key area responsible for hunger and fullness sensations.^264–266^ The hypothalamus contains various neurons that respond to different nutritional signals and hormones, like GLP-1, to regulate food intake.^265,266^ When GLP-1 binds to its receptors in the hypothalamus, it activates specific neurons, including those that promote satiety (POMC/CART neurons) and inhibits those that induce hunger (NPY/AgRP neurons), thereby enhancing the feeling of fullness and reducing food intake.^233,267^

GLP-1 can slow down gastric emptying through its effects on smooth muscles and the nervous system in the gastrointestinal tract.^264,268^ Released at the gut’s end, GLP-1 acts on its receptors in the gastrointestinal tract, leading to reduced gastric muscle contractions and extended food retention in the stomach.^264,269^ Furthermore, GLP-1 can slow down gastric emptying by activating the vagus nerve, a part of the autonomic nervous system crucial for regulating the activities of the gastrointestinal tract.^268^ When activated, the vagus nerve can reduce the contraction of the stomach, thereby slowing down food emptying.^269^ The prolonged retention of food in the stomach enhances the feeling of fullness, naturally reducing overall food intake.^270,271^ Additionally, slower gastric emptying helps stabilize postprandial blood glucose levels. Reducing food intake and extending satiety are significant for weight management and loss. By regulating gastric emptying, GLP-1 not only aids in short-term appetite control but may also contribute positively to long-term energy balance and weight management.^176^

## GLP-1RAs and diseases

As a rising star in recent years, GLP-1RAs are not only effective in metabolic diseases but also play a role in non-metabolic disorders, affecting multiple systems including the musculoskeletal, nervous, cardiovascular, and digestive systems, and can even have implications in oncological diseases.

### Musculoskeletal system

Disorders associated with the musculoskeletal system often cause painful swelling and permanent damage in the joints of the body, especially the hips, knees and thumbs. These diseases may affect more people in today’s aging society. Because of the close relationship between risk factors in human metabolism and the expression of GLP-1R in the musculoskeletal system, GLP-1RAs may have great potential in the treatment of many diseases of the musculoskeletal system.^15,272^

#### GLP-1RAs and joint disorders

##### GLP-1RAs in OA

GLP-1R expression was detected via immunohistochemistry in articular chondrocytes from both normal and osteoarthritic individuals.^273^ The primary outcome of GLP-1R expression involves suppressing the release of cytokines into the synovial fluid, leading to a reduction in inflammation.^274,275^ This, in turn, diminishes additional downstream effects, including oxidative stress, the secretion of pro-degradative substances, modifications to cell phenotype (hypertrophy, M1/M2 macrophage phenotype, fibrosis), and damage or deterioration of joint cells (apoptosis, senescence).^15,276^

The activation of GLP-1R is linked to decreased NF-κB pathway activity Treatment with GLP-1RAs can effectively mitigate chondrocyte apoptosis caused by endoplasmic reticulum stress and alleviate the associated inflammatory response.^277–280^ This effect is accomplished through the inhibition of JNK, NF-κB, and other relevant signaling pathways.^273^ Moreover, GLP-1RAs could decelerate the progression of OA and mitigate pathological damage in a rat OA model.^273^ In addition, in a rat model of inflammatory OA induced by monoiodoacetic acid (MIA), researchers have shown that the activation of GLP-1R triggers the PKA/CREB signaling pathway, leading to a reduction in cartilage inflammation.^281^

Inflammation in OA is closely related to the activation of macrophages.^282,283^ These cells accumulate in the synovial membrane and subchondral bone, releasing pro-inflammatory cytokines such as TNF-α, IL-1β, and IL-6, which promote the degradation of joint cartilage and the inflammatory response.^284–286^ Macrophages express GLP-1Rs, which are involved in regulating their inflammatory responses. Binding of GLP-1 or GLP-1RAs to these receptors can activate the cAMP/PKA signaling pathway, affecting key transcription factors like NF-κB.^287,288^

This binding initiates typical GPCR signaling, leading to G protein activation and increased intracellular cAMP.^289^ The rise in cAMP activates PKA, a versatile protein kinase that phosphorylates various target proteins.^290–292^ In macrophages, PKA regulates gene expression by phosphorylating transcription factors like CREB.^293^ CREB activation can promote the expression of anti-inflammatory genes while inhibiting inflammatory genes.^294,295^ In inflammation regulation, NF-κB is a key transcription factor.^296–298^ Typically, NF-κB binds to its inhibitor IκB in the cytoplasm.^297,299^ When IκB is phosphorylated and degraded, NF-κB can move to the nucleus, activating multiple inflammation-related genes.^300,301^ This not only affects the intracellular signaling of macrophages but also their response to the external environment, including their effects on chondrocytes and other synovial cells.^302–305^

GLP-1R signaling, through PKA, inhibits this process, possibly by promoting IκB stability or inhibiting its phosphorylation, thus reducing NF-κB activation and nuclear translocation.^8,281^ Through these mechanisms, GLP-1R signaling influences the production of inflammatory factors in macrophages.^306^ For example, the reduction in TNF-α, IL-1β, and IL-6 helps regulate local and systemic inflammatory responses.^307,308^ Additionally, GLP-1R signaling may affect other macrophage functions, such as phagocytosis, migration, and cell survival.^281^

By reducing macrophage-mediated inflammatory responses, GLP-1RAs have the potential to alleviate symptoms of OA, including pain and joint stiffness.^281,309^ Moreover, reducing inflammation may slow down the degradation of joint cartilage, providing long-term joint protection.^310,311^ GLP-1RAs also enhance autophagy, a process of cellular clearance of damaged and outdated components.^13,312^ Regulation of autophagy may help remove harmful proteins and other cellular debris accumulated under inflammatory conditions, which is potentially important for maintaining the health of joint tissues.^313,314^

In one study, OA was induced in rats by injecting MIA into the knee joint, mimicking the pathological changes of human OA.^315^ Subsequently, rats were treated with liraglutide through subcutaneous injection to observe its therapeutic effect on OA.^315^ The expression levels of GLP-1R, PKA/CREB signaling pathway components, and inflammation-related proteins (such as TNF-α, IL-1β, and IL-6) in the rat knee cartilage tissue were measured using Western blot and immunoprecipitation techniques.^315^ The results showed that, in the OA rat model, liraglutide could activate the PKA/CREB signaling pathway and inhibit the inflammatory response through this pathway, thereby alleviating OA symptoms.^316^ These findings provide scientific evidence for developing new OA treatment strategies, confirming the potential of GLP-1 agonists in treating OA.^315–317^

GLP-1R is expressed in human monocyte-derived macrophages and the mouse macrophage cell line RAW264.7.^279,318^ c-Jun N-terminal kinase (JNK) is a mitogen-activated protein kinase (MAPK) involved in regulating cellular stress responses and inflammation.^318^ Signal Transducer and Activator of Transcription 3 (STAT3) is a transcription factor involved in cell growth, differentiation, and inflammatory responses.^319–321^ Researchers found that activation of GLP-1R could play a key role in regulating macrophage polarization by adjusting the phosphorylation levels of JNK and STAT3.^318,322^ Macrophages have two polarization states, M1 typically has pro-inflammatory properties, while M2 has anti-inflammatory properties.^323–325^ Specifically, activation of GLP-1R leads to an increase in cAMP levels, which in turn activates PKA, a widely regulating enzyme of cellular functions, capable of phosphorylating a variety of target proteins, thereby initiating the PKA/CREB signaling pathway.^326,327^ This not only prevents the phosphorylation of JNK but also promotes the phosphorylation of STAT3, aiding the shift of macrophages to an anti-inflammatory M2 phenotype.^327^ In the inflammatory environment, the M1 to M2 shift promoted by GLP-1 is crucial for reducing the expression of inflammatory factors such as IL-6, TNF-α, and iNOS.^8,15,328^

Chondrocytes are the only cell type in joint cartilage, responsible for synthesizing and maintaining the integrity of the cartilage matrix.^329,330^ In degenerative joint diseases like OA, the metabolic balance of chondrocytes is disrupted, leading to the overproduction of degrading enzymes and inflammatory mediators, including iNOS, MMP-13, and ADAMTS5 (a disintegrin and metalloproteinase with thrombospondin motifs 5), which participate in cartilage degradation and inflammatory processes.^331^ iNOS (inducible nitric oxide synthase) produces nitric oxide (NO) during inflammation, modulating signal transduction; MMP-13 (matrix metalloproteinase-13) is involved in cartilage degradation;^331^ ADAMTS5 is closely related to cartilage damage and inflammation.^332–334^ The NO produced by iNOS, as a free radical, can regulate intracellular signal transduction and modulate the inflammatory response.^335,336^ The expression of iNOS is primarily activated by the NF-κB pathway, which is activated and translocated to the nucleus upon inflammatory stimulation (such as bacterial endotoxins or pro-inflammatory cytokines), thus increasing the transcription and expression of iNOS.^337–339^ GLP-1 can exert its anti-inflammatory effects by activating its receptor, GLP-1R.^339^ When GLP-1 binds to GLP-1R, it activates the cAMP signaling pathway, leading to increased cAMP levels.^1,340^ The rise in cAMP further activates PKA, which can inhibit the NF-κB signaling pathway, reducing the production of inflammatory factors such as iNOS and other inflammation-related proteins.^12,340,341^ The expression of MMP-13 is regulated by IL-1β and TNF-α. These factors promote the transcription of the MMP-13 gene by activating the MAPK and NF-κB signaling pathways.^342–344^ The GLP-1R signaling pathway may also affect the expression of MMP-13 and ADAMTS5, by reducing the signal transduction caused by IL-1β, thus decreasing their synthesis.^345,346^ GLP-1 may also reduce the expression of cartilage-degrading enzymes by inhibiting the MAPK pathway or activating anti-inflammatory pathways such as PI3K/Akt, thereby alleviating cartilage damage.^206,347^

In another study, treatment of primary mouse chondrocytes with liraglutide reduced the mRNA expression levels of iNOS, MMP-13, and ADAMTS5, leading to a decrease in the secretion of inflammatory markers, including NO, prostaglandin E, and IL-6.^348^ Similarly, in human chondrocytes stimulated by TNF, GLP-1 analogs (such as liraglutide) showed an anti-catabolic effect, reducing the mRNA expression of MMP-3, MMP-13, and ADAMTS5.^349,350^ At the same time, the levels of two important components of the cartilage matrix, proteoglycans (a large molecule and a major component of the cartilage matrix) and type II collagen (the main structural protein of cartilage), increased.^349,350^ This change suggests that liraglutide not only inhibits inflammation and cartilage degradation but may also promote the synthesis and accumulation of cartilage matrix, thereby helping to protect and repair joint cartilage.^273^ Furthermore, a study using the anterior cruciate ligament transection (ACLT) rat model further confirmed that subcutaneous injection of liraglutide at 50 μg/kg/day, whether for 3 weeks or 6 weeks, could reduce OARSI scores, highlighting the potential of liraglutide in treating joint degeneration.^273^

##### GLP-1RAs and RA

The role of GLP-1RA was also investigated in RA, which is characterized by chronic inflammation of the synovium and joint destruction. In fibroblast-like RA synoviocytes, the administration of lixisenatide resulted in a reduction in the inflammatory response.^347^ This was achieved by decreasing the expression of proinflammatory cytokines such as tumor necrosis factor (TNF), interleukin-6 (IL-6), and interleukin-8 (IL-8).^15,351,352^ Moreover, lixisenatide has been shown to inhibit matrix metalloproteinase (MMP) activity and effectively block various cell signaling pathways, including the JNK, activator protein-1, and NF-κB pathways.^353^ These findings confirm that in the synovium, GLP-1R is expressed on two different cell types, macrophages and fibroblast-like synoviocytes, which are specialized cells distributed within the synovial intima and subintima, and these cell types play important roles in hyaluronic acid synthesis, metabolite processing, and clearance of matrix degradation fragments.^354^

#### GLP-1RAs and musculoskeletal health

##### GLP-1RAs and bone

The quality of bone depends on bone metabolism, and the main factors affecting bone metabolism include osteoclasts, osteoblasts, and calcitonin.^355–357^ According to most related studies, osteoclasts and osteoblasts are indispensable for bone remodeling, and bone resorption and bone formation are mediated by osteoclasts and osteoblasts, respectively.^358^ GLP-1R is present in bone marrow stem cells (BMSCs),^15,359^ osteoblasts,^15^ osteocytes,^360^ and osteoclasts,^361^ and GLP-1RAs have the potential to impact these cells.

**GLP-1RAs and osteoclasts** The greater degree of bone degradation observed in mice lacking GLP-1R indicates that GLP-1R signaling suppresses osteoclast differentiation and bone resorption.^362^ In an experimental study in which osteoclast formation and bone resorption were induced in mice through lipopolysaccharide (LPS) administration, researchers discovered that simultaneous treatment with exendin-4 resulted in a significant decrease in the number of osteoclasts, the proportion of bone resorption pits, and the levels of the bone resorption marker CTX compared to injection with LPS alone.^363^ According to previous reports, exendin-4, a GLP-1RA, has the potential to inhibit LPS-induced osteoclast formation and bone resorption in vivo.^363,364^ This inhibition is believed to occur through the suppression of LPS-induced TNF-α production in macrophages.^15^ Studies have indicated that GLP-1R KO mice exhibit greater numbers of osteoclasts and greater bone resorption than wild-type controls.^364^ Additionally, µCT analysis revealed that, compared with their wild-type counterparts, hyperlipidemic rats treated with subcutaneous GLP-1 for 3 days exhibited increased bone mass in the femur and vertebrae.^365^

Diabetic patients have a higher risk of fractures than does the general population.^366^ Mice with type 1 diabetes (T1D) exhibit a reduction in bone mineral density (BMD) and compromised microstructural integrity.^367^ The administration of liraglutide also impeded osteoclastic bone formation, thereby inhibiting bone resorption and exerting protective effects on bone health in T1D mice.^367^ Specifically, liraglutide, both alone and in combination with insulin, effectively suppressed the formation of osteoclasts. This effect is achieved by reducing the expression of Trem2 and NFATc1 and downregulating the expression of CTSK and TRAP to inhibit bone resorption activity. These findings provide further evidence for the impact of GLP-1RAs on osteoclastic bone resorption.^367^ A study involving 12-week-old ovariectomized mice revealed that administering liraglutide for 4 weeks effectively prevented the loss of trabecular bone. The analysis of bone tissue morphology revealed that there were no alterations in the rate of bone formation or in the levels of calcitonin or sclerostin in these mice. These findings suggest that liraglutide specifically reduces bone resorption without influencing bone formation.^368^

Since GLP-1R is expressed in thyroid C cells and GLP-1 directly stimulates the secretion of calcitonin, which is a potent inhibitor of bone resorption in osteoclasts, GLP-1 may contribute to the nutrient-mediated reduction in bone resorption.^369,370^ Genetic disruption of GLP-1R signaling leads to cortical osteopenia and heightened bone fragility, primarily caused by increased bone resorption by osteoclasts. This change was accompanied by a decrease in thyroid calcitonin expression. Furthermore, the administration of exogenous GLP-1 resulted in elevated calcitonin expression in the thyroids of normal (wild-type) mice.^362^ The administration of calcitonin successfully reduced the levels of urinary deoxypyridinoline in GLP-1R knockout mice. Additionally, treatment with GLP-1RA and exendin-4 increased the expression of the calcitonin gene in the thyroids of normal (wild-type) mice. These findings provide evidence that the regulatory influence of endogenous GLP-1R signaling on bone resorption is likely mediated through pathways that involve calcitonin.^362^ Considering the expression of the GLP-1R in thyroid C cells and the ability of GLP-1 to stimulate calcitonin secretion through a cAMP-mediated mechanism in vitro, it is plausible that calcitonin plays a role in the alterations in bone metabolism observed in GLP-1R-treated animals.^369,370^ Later, quantitative real-time PCR analysis demonstrated that the administration of exendin-4, a GLP-1RA, resulted in significant upregulation of thyroid calcitonin mRNA levels in wild-type mice.^362^

**GLP-1RAs and Osteoblasts** Osteoblasts arise through the differentiation of mesenchymal stem cells and play a crucial role in the process of bone formation. Stimulating GLP-1R in BMSCs triggers the buildup of nuclear β-catenin, which, in turn, activates osteogenic genes by binding with TCF7L12.^371^ In osteoblasts, the administration of GLP-1 and GIP incretins suppressed the excessive expression of the pro-degradative enzymes MMP-3 and MMP-13 induced by IL-1β stimulation.^372^ In vitro, GLP-1 disrupts the ability of osteoblasts to survive and differentiate by triggering the activation of c-Fos, which is a proto-oncogene.^373^ In fact, when BMSCs are exposed to exendin-4, the expression of genes related to bone development factors such as Runx and Osterix, as well as genes responsible for producing the bone matrix such as Balp and Bglap, is upregulated. Moreover, stimulation of GLP-1R in BMSCs results in the accumulation of β-catenin in the cell nucleus. This accumulation facilitates the binding of β-catenin to TCF7L12, triggering the activation of genes associated with osteogenesis.^371^

**Fig. 3:**
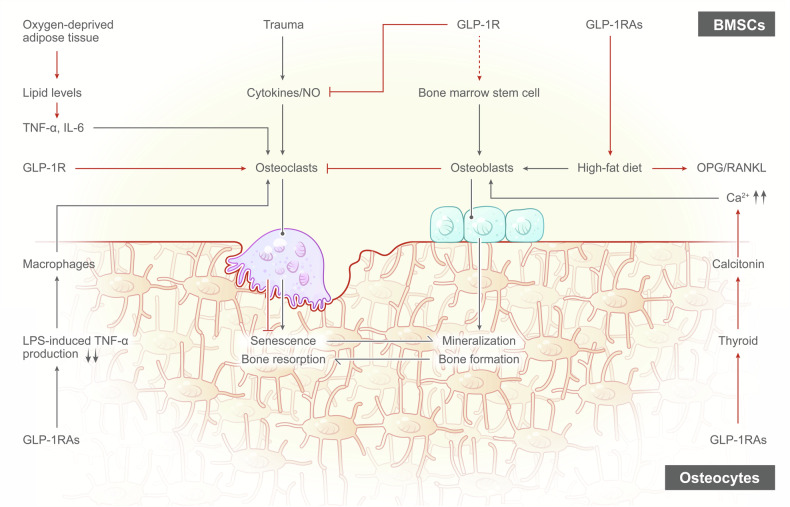
The Effects of GLP-1RAs on osteoclasts and osteoblasts. GLP-1RAs aid in weight loss by regulating the gut-brain axis and interacting with leptin, while weight loss can alleviate the harmful effects of obesity on the body, particularly in knee OA, by reducing joint loading and inflammation. Obesity disrupts bone metabolism and leads to increased bone resorption, but GLP-1RAs can inhibit this damage and improve bone health by increasing the OPG/RANKL ratio, reducing osteoclast activity, and promoting bone formation

In a study examining the effects of GLP-1RA on osteoporosis induced by ovariectomy in aged rats, administering exendin-4 for 16 weeks prevented deterioration of the trabecular microarchitecture and increased bone strength. This was achieved by inhibiting bone resorption through an increase in the OPG/RANKL ratio and promoting bone formation by enhancing the expression of osteoblast-specific transcription factors.^374^ Exendin-4 has also been shown to stimulate osteoblast activity and mitigate bone loss in an ovariectomized mouse model.^374^ Liraglutide can directly enhance bone formation in the MC3T3-E1 osteoblastic cell line. This effect is achieved through the activation of signaling pathways such as the ERK1/2, PI3K/AKT, and cAMP/PKA/β-cat-Ser675 pathways, which are mediated by GLP-1RAs.^375^ (Fig. 3).

##### GLP-1RAs and muscle

**GLP-1RAs and Muscle Atrophy** In previous experiments, exendin-4 (Ex-4) inhibited the expression of myostatin (MSTN), atrophy-factor F-box only protein 32 (atrogin-1) and muscle ring finger protein 1 (MuRF-1) in dexamethasone-treated C2C12 myotubes.^376–378^ In a dexamethasone-induced muscle atrophy model, Ex-4 ameliorated muscle atrophy by inhibiting muscle atrophy factor and enhancing myogenic factors (MyoG and MyoD), thereby increasing muscle mass and function. In the muscle atrophy mouse model, Ex-4 also increased muscle mass and muscle fiber size and improved muscle function. In addition, treatment with the long-acting GLP-1RA duraglutide restored muscle mass and function in DBA/2J-mdx mice.^379^

GLP-1RAs, particularly PF1801, have demonstrated effective relief in inflammatory myopathies, such as polymyositis (PM), through preclinical studies.^376,380^ The therapeutic effects of PF1801 are primarily achieved by modulating several key proteins that play central roles in inflammation and cellular metabolism.^380^ Firstly, the expression of GLP-1R is enhanced in the inflamed muscle fibers under pathological conditions, revealing the critical role of GLP-1R in regulating the muscle’s response to inflammation.^381^ PF1801 activates these receptors, initiating a series of biological responses that influence the inflammatory state of muscle cells, thereby alleviating inflammation. Next, PF1801 exerts its effects by activating AMP-activated protein kinase (AMPK).^382^ As a central node in energy sensing and metabolic regulation, the activation of AMPK helps maintain cellular energy balance and prevents cell death due to energy depletion.^383,384^ Importantly, the activation of AMPK reduces the expression of phosphoglycerate mutase 5 (PGAM5), which plays a promotive role in cell necrosis by contributing to mitochondrial dysfunction and the production of reactive oxygen species (ROS).^385–387^ Thus, by inhibiting PGAM5, AMPK suppresses necrosis, reduces ROS accumulation, and mitigates oxidative stress.^385,387^ PF1801 displays its anti-inflammatory effects by lowering levels of inflammatory mediators such as TNFα, IL-6, and HMGB1, and enhances the cell’s antioxidant capability by upregulating molecules like Nfe2l2, Hmox1, Gclm, and Nqo1, which further improves cellular defense against oxidative stress and protects them from further damage.^382^ Through this sophisticated molecular regulation, PF1801 not only alleviates inflammation and necrosis in muscle fibers but also enhances the energy and antioxidant status of muscle cells.^382^ This contributes to maintaining muscle strength and reducing inflammation. This comprehensive change reflects how alterations in the expression of individual key proteins can impact the entire metabolic and inflammatory pathways, thereby improving disease conditions and enhancing therapeutic efficacy.^376^

**GLP-1RAs in Enhancing Exercise Endurance** Studies indicate that acute exercise and short-term endurance training significantly increase GLP-1 secretion in mice. In endurance-trained men, GLP-1 plasma concentrations are elevated immediately at 30 and 45 minutes after exercise.^388^ To confirm the role of GLP-1 in enhancing physical endurance and the possible mechanisms involved, an in vivo AAV-mediated GLP-1 overexpression model and an in vitro siRNA-mediated AMPK knockdown model were generated. We demonstrated that GLP-1 enhances physical endurance by inducing skeletal muscle remodeling, which may be mediated by GLP-1R/AMPK signaling.^389^ Overall, GLP-1 secretion is induced by exercise. Overexpression of GLP-1 in skeletal muscle can improve endurance. These results suggest that GLP-1 may improve exercise endurance in mice by enhancing skeletal muscle glycogen synthesis and glucose uptake. Mitochondrial content and function in skeletal muscle are regulated by GLP-1.^389^ The interaction between GLP-1 and its receptor GLP-1R initiates the AMPK signaling cascade within skeletal muscle tissue. This initiation precipitates a multitude of alterations in the cellular milieu, notably the augmentation of mitochondrial biogenesis-a mechanism responsible for the genesis of new mitochondria. Furthermore, GLP-1 augments mitochondrial efficacy, as manifested by the ameliorated oxidative metabolism of muscle tissue. This enhancement is demonstrable through an increase in mitochondrial DNA content, the upregulation of genes integral to mitochondrial biogenesis, and the increased expression of proteins pivotal in oxidative phosphorylation. These cellular transformations contribute significantly to enhanced endurance during exercise, thereby underscoring the critical role that GLP-1 plays in the regulation of mitochondrial function and content in skeletal muscle.^389^

#### GLP-1RAs and fat metabolism

GLP-1RAs work via numerous mechanisms that contribute to weight loss, one of the most well-known of which is the gut-brain axis.^212,213,390^ Within this axis, GLP-1 functions by acting on both the gut and the brain.^391^ Furthermore, the combination of GLP-1-mediated signaling and the adipocyte hormone leptin has recently garnered increased interest. Notably, leptin may serve as a crucial biological signal for GLP-1, working in synergy, to decrease food intake and body weight. The effects of the leptin-GLP-1 interaction may be governed by intracellular signaling pathways, including those involving phosphorylated STAT3 and PTP1B.^15,392^

In Wistar rats, a diet high in fat was found to lead to a decrease in the ratio of OPG/RANKL, which resulted in increased bone resorption and ultimately a reduction in bone mass. The administration of GLP-1RA or exendin-4 to rats fed a high-fat diet resulted in an increase in the OPG/RANKL ratio and a reduction in the degree of bone loss. It was observed that the treatment of rats on a high-fat diet with exendin-4 resulted in a decrease in the number of osteoclasts and the area of eroded surfaces, while there was an increase in the osteoid area, bone mass, and trabecular bone volume. These effects were compared to those of untreated controls that were also maintained on a high-fat diet.^393^ As a result, GLP-1RAs could mitigate the detrimental effects of hyperlipidemia-induced skeletal defects, leading to an improved prognosis in individuals with OA.

**Fig. 4:**
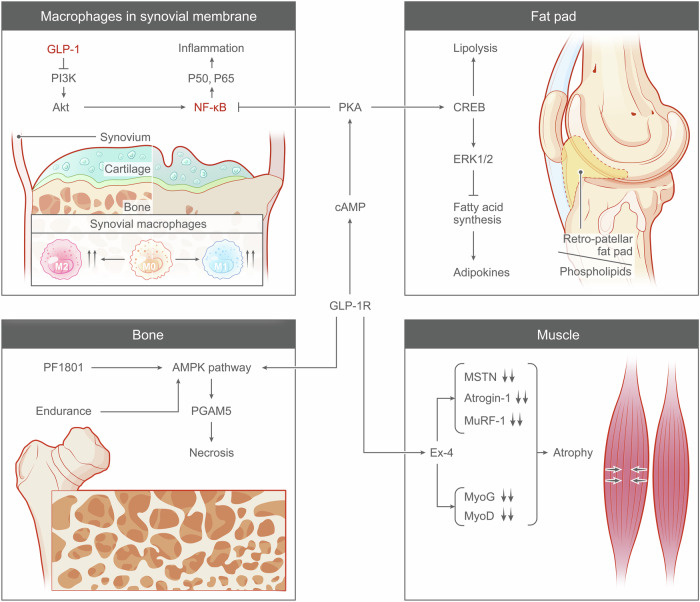
The Effects of GLP-1RAs on Musculoskeletal System. GLP-1RAs inhibit chondrocyte apoptosis, reduce inflammation, and protect articular chondrocytes in OA and RA through various mechanisms, including suppressing cytokine release, inhibiting the NF-κB pathway, and reducing inflammation-related gene expression. They have shown to reduce the inflammatory response by decreasing the expression of proinflammatory cytokines and inhibiting matrix metalloproteinase activity and cell signaling pathways. GLP-1R expression is found in macrophages and fibroblast-like synoviocytes, which are important for maintaining synovial fluid homeostasis. GLP-1R signaling stimulates calcitonin secretion, which inhibits bone resorption, and disruption of GLP-1R leads to increased bone resorption and decreased calcitonin expression. GLP-1RAs have pleiotropic effects on skeletal muscle, including inhibiting muscle atrophy, preserving muscle strength, and enhancing exercise endurance, through GLP-1R-mediated signaling pathways. GLP-1RAs can influence the composition of the phospholipid layer on cartilage, leading to beneficial effects on joint health and potentially facilitating repair of existing damage in individuals with OA

On the surface of the cartilage, there is a layer of special phospholipids. When a joint bears weight, it functions as a lubricant, playing a crucial role in enabling the joint to continue operating efficiently and smoothly. Altering the composition of this phospholipid layer can impact the functioning of the corresponding joint. For instance, changes in the composition of this phospholipid layer have been observed by researchers, along with their detrimental effects on the bones and joints of individuals with OA.^394^ Surprisingly, GLP-1RAs can influence the phospholipid structure and cytokines surrounding joints, leading to beneficial transformations that safeguard joints and even facilitate partial repair of any existing damage.^395^ (Fig. 4).

### GLP-1RAs in nervous system

In the nervous system, GLP-1 can decrease oxidative stress and inflammatory responses, potentially through reducing the generation of ROS and decreasing the expression of inflammatory cytokines. Activation of its receptor by GLP-1 can promote the phosphorylation of CREB, subsequently fostering the expression of genes related to neuronal survival and regeneration.^396–399^

#### GLP-1RAs and nerve cells

GLP-1 can modulate peripheral nerves through the ERK (extracellular signal-regulated kinase) signaling pathway, reducing the occurrence of neurological dysfunction.^400^ Researchers have discovered that GLP-1RAs exhibit a noticeable increase in the level of phosphorylated ERK1/2 within the sciatic nerve of diabetic rats. This observation led them to speculate that GLP-1 analogs might possess distinct neurotrophic properties and exert protective effects specifically on the nerve.^71,395,401–403^

Astrocytes, the most abundant cell type in adult brain nerve tissue, have recently been recognized for their pivotal role in regulating glucose and energy homeostasis.^404–406^ These cells not only respond to signals from leptin and insulin but also adapt to alterations in brain metabolism to accommodate behavioral changes by controlling glucose transport.^406,407^ Furthermore, astrocytes express GLP-1R, which has been found to be crucial for their proper functioning.^408^ Studies have shown that the loss of GLP-1R in astrocytes can impair mitochondrial integrity, leading to the dysfunction and inhibition of glucose uptake and β-oxidation.^407^ GLP-1 inhibits glucose uptake in astrocytes and promotes beta-oxidation, which is essential for regulating energy balance in the brain and maintaining mitochondrial integrity.^406^ When GLP-1R is knocked out in astrocytes, it activates an integrated stress response, which affects the overall metabolic state by increasing the production of FGF21.^405^ This suggests that signaling through GLP-1R in astrocytes is crucial for maintaining the metabolic stability and functionality of the cells.^406^ FGF21 is recognized as a stress response factor that addresses mitochondrial dysfunction in cells. In astrocytes lacking GLP-1R, the increase in FGF21 is associated with improved systemic glucose homeostasis and memory formation.^406^ This indicates that FGF21 not only plays a role in cellular stress responses but also has a significant role in regulating brain function and systemic metabolism.^406^ Therefore, endogenous GLP-1R signaling in astrocytes plays a critical role in maintaining mitochondrial homeostasis and is dependent on FGF21 to effectively regulate glucose metabolism.^406,407^

GLP-1RAs can alleviate neuroinflammation by acting on the nervous system, thereby offering additional relief from pain in patients.^6,409,410^ During a previous experiment, researchers induced pain and observed symptoms of cognitive impairment in rats through spinal nerve ligation. Then, the researchers administered exendin-4 via intrathecal injection to the experimental rats and observed a reduction in pain sensitivity, alleviation of neural inflammation, and suppression of inflammatory factors such as IL-1β, TNF-α, and iNOS. This finding suggests a direct correlation between the analgesic effects of GLP-1R signaling and its Anti-neuroinflammatory activity.^411^ In another study, 3D fluorescence microscopy was used to eliminate proteins within chondrocytes in the subchondral bone of cartilage, resulting in bone transparency and the acquisition of high-resolution 3D images. By utilizing this methodology, researchers have revealed that GLP-1RAs can exhibit affect the axon terminals of sensory neurons. Ultimately, it was revealed that cholinergic fibers are present within the subchondral bone of individuals with OA, and the release of acetylcholine (Ach) triggered by vagus nerve stimulation plays a prominent role in combating inflammation across various diseases while also providing pain relief.^412^ Notably, GLP-1RAs, including exendin-4, have been shown to attenuate microglial activation, resulting in a reduction in the expression of proinflammatory cytokines such as TNF-α and IL-1β. By modulating inflammatory responses, GLP-1RAs can help prevent the degeneration of dopamine-producing cells.^413^ GLP-1RAs play a neuroprotective role in neurons by facilitating neuronal survival and promoting neuronal growth, thereby preserving the structural and functional integrity of synapses.^6,14^ Furthermore, GLP-1 signaling indirectly enhances and restores the insulin signaling pathway in neurons, leading to a reduction in the phosphorylation of insulin receptor substrates and the burden caused by monomeric α-synuclein.^414^ Together, these effects contribute to the protection of dopaminergic neurons (source). Exenatide has been shown to mitigate the phosphorylation of insulin receptor substrates and the accumulation of monomeric α-synuclein.^414^ By activating the GLP-1 signaling pathway, exenatide exhibits neuroprotective effects and safeguards dopaminergic neurons.^5^

The most well-known mechanism through which GLP-1RAs affect weight loss is through the endocrine pathway, but its role in the nervous system should not be overlooked.^415–417^ GLP-1RAs can exert their local effects by activating vagal dendritic terminals that innervate the gut.^418–420^ This activation holds the ability to modulate food consumption by reducing food intake and conveying signals of satiety to the brain.^391^ GLP-1 and GLP-1 analogs exert their effects on food intake and body weight through a myriad of neural substrates, encompassing several hypothalamic nuclei (including the arcuate nucleus of the hypothalamus, periventricular hypothalamus, and lateral hypothalamic area), hindbrain nuclei (such as the parabrachial nucleus and medial nucleus tractus solitarius), the ventral subregion of the hippocampus (vHP), and nuclei embedded within the mesolimbic reward circuitry (including the ventral tegmental area, VTA, and nucleus accumbens, NAc).^421,422^ Remarkably, GLP-1R activation in certain nuclei (such as the VTA, NAc, and vHP) elicits reductions in food intake and body weight without concurrent nausea responses.^392^

#### The relationship of inflammatory response and neurodegenerative disease

In the research conducted by Daniel J. Drucker’s team, the mechanism of anti-inflammatory action of GLP-1RAs was explored, revealing a key role for the CNS in regulating this anti-inflammatory effect.^423^ The study began by inducing inflammation in mice through the injection of various Toll-like receptor (TLR) agonists and then assessed the inflammation by measuring plasma tumor necrosis factor-alpha (TNF-α) levels.^423^ It was observed that the GLP-1RA, exendin-4, significantly reduced the TNF-α levels caused by various TLR agonists, indicating that exendin-4 can lower the TNF-α levels induced by multiple TLR agonists.^423^ The research further demonstrated that this anti-inflammatory effect was not mediated by GLP-1R in the blood or endothelial cells but required the GLP-1R in the CNS.

Additionally, to simulate sepsis caused by polymicrobial infection and assess the impact of GLP-1RAs, the study utilized a cecal content injection method. It was found that semaglutide, a long-acting GLP-1RA, could improve symptoms caused by sepsis, reduce body temperature, and decrease the bacterial load in multiple organs, along with the levels of inflammatory factors in plasma and lungs.^423^ These findings further confirmed the central role of the CNS in regulating the anti-inflammatory effects of GLP-1RAs, highlighting the potential application of GLP-1RAs in anti-inflammatory treatment.

The study also discovered the roles of α1-adrenergic and δ- and κ-opioid receptors in this process. Specifically, blocking the α1-adrenergic receptors with prazosin or the opioid receptors with nalbuphine interfered with the ability of GLP-1RAs to reduce plasma TNF-α levels, indicating the critical importance of these pathways in the anti-inflammatory effects mediated by GLP-1 activation. The α1-adrenergic receptors, found on the surface of cells, facilitate various physiological responses, including regulating vascular contraction and heart rate. The use of prazosin, an α1-adrenergic receptor blocker, showed that the ability of GLP-1RAs to reduce TNF-α in plasma was disrupted, signifying the vital role of α1-adrenergic receptors in the anti-inflammatory action of GLP-1RAs. Opioid receptors, which are associated with pain regulation, mood, and immune responses, include δ and κ types. The use of nalbuphine, a blocker of δ- and κ-opioid receptors, also disrupted the effect of GLP-1RAs on reducing TNF-α levels, highlighting the essential role of these opioid receptors in the anti-inflammatory effects mediated by GLP-1RAs.^423^

Furthermore, the study emphasized that neurons in specific brain regions, such as the hindbrain and hypothalamus, co-express GLP-1R along with α1-adrenergic and δ-opioid receptors, suggesting a localized mechanism within the CNS that could coordinate peripheral anti-inflammatory responses. This co-expression indicates a mechanism within the CNS allowing these neurons to sense and respond to the peripheral inflammatory state. Through the interaction of these receptors, the brain can receive signals of peripheral inflammation and respond through neural signaling, thereby modulating or alleviating the inflammatory response. This receptor co-expression on neurons in specific brain regions may enable the brain to finely regulate the body’s response to inflammation. For instance, when peripheral tissues become inflamed, the related signals might be transmitted to the brain through these receptors on the neurons, and the brain could then respond to these signals via neural pathways, thus regulating the level of peripheral inflammation.^423^

#### GLP-1RAs and AD

AD is a progressive and irreversible neurodegenerative disorder characterized by an unclear etiology and pathogenesis.^424^ In AD, GLP-1(7-36) amide inhibits IL-1β transcription and prevents cognitive dysfunction, amyloid precursor protein synthesis, and cell death. It also enhances learning and memory by promoting long-term potentiation (LTP).^425,426^ In a murine model of AD, the administration of GLP-1RAs effectively reduced the levels of pathological markers associated with AD. These markers include oligomeric antibodies and amyloid plaque load. Furthermore, GLP-1RAs have been shown to attenuate microglial activation and improve memory-related behaviors.^71^ Moreover, GLP-1RAs have demonstrated considerable therapeutic promise in animal models of both PD and AD.^427–429^ The compound NLY01 is particularly effective at attenuating the activity of proinflammatory microglia and preventing the transformation of astrocytes to a reactive phenotype. This activity is instrumental in safeguarding hippocampal neurons from the deleterious impacts of glutamate-induced excitotoxicity and hypoxic conditions. In the context of AD, where neuroinflammation and neurotoxicity are prominent pathological hallmarks, the modus operandi of NLY01 offers a potential therapeutic avenue to mitigate analogous pathological mechanisms inherent to this neurodegenerative condition.^430^

#### GLP-1RAs and PD

PD is a chronic neurodegenerative disorder that affects the CNS and is the second most prevalent neurodegenerative disease worldwide.^14^ GLP-1RAs are promising pharmacological agents for treating PD due to their potential to preserve the integrity and function of dopaminergic neurons. In addition to its effects on metabolic regulation, GLP-1, when synthesized within the brain, exhibits neuroprotective properties.^397,427,431^ A clinical trial titled “Liraglutide in Early Parkinson’s Disease” was published in the New England Journal of Medicine, exploring the effects of liraglutide on patients with early-stage PD diagnosed within the last three years.^432^ The research was a 14-month Phase II double-blind randomized controlled trial. The results showed that liraglutide had a modest positive effect on improving motor function and performed well in terms of safety and tolerability, although there were some manageable gastrointestinal side effects.^432^ The study emphasizes the need for further research into the potential benefits and risks of liraglutide in patients at different stages of PD.^432^

#### GLP-1RAs and addictive disorders

Researchers have discovered that semaglutide has the ability to reduce both recurrent alcohol consumption and overall alcohol intake in rats by more than 50%.^433^ Specifically, alcohol-dependent rats were administered semaglutide, which resulted in a significant reduction in their alcohol consumption.^433^ Further investigation into the mechanism underlying the alcohol-reducing effects of semaglutide suggested that this effect may involve the modulation of alcohol-induced rewards and punishments within the brain. Researchers have also shown that semaglutide impacts the reward and punishment systems in the brains of mice, particularly in the nucleus accumbens region, which is part of the limbic system.^433^ It is believed that alcohol activates the brain’s reward and punishment system, triggering the release of dopamine, a neurotransmitter associated with pleasure and reward, both in humans and animals. However, the administration of semaglutide appeared to block this process, potentially leading to a diminished alcohol-induced reward and punishment response within the body.^433^ Remarkably, compared with untreated rats, treated rats exhibited a significant reduction in alcohol intake, which was reduced by half.^433^ These findings highlight the potential therapeutic efficacy of semaglutide in mitigating alcohol consumption.^434^

**Fig. 5:**
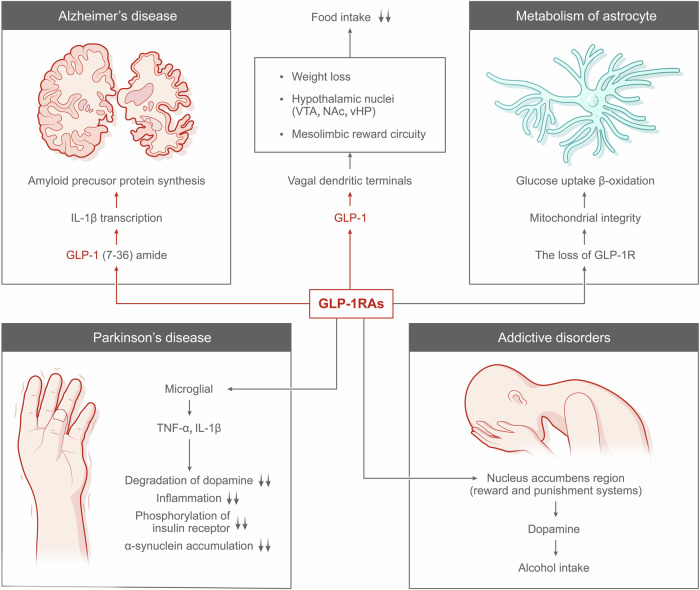
The effects of GLP-1RAs on nervous system. GLP-1RAs have diverse effects, including alleviating neuroinflammation and pain in OA, reducing food intake and improving body weight, protecting peripheral nerves, maintaining astrocyte function and metabolic homeostasis, and showing potential therapeutic benefits in AD and PD. Semaglutide, a diabetes drug, can also reduce alcohol consumption by modulating the brain’s reward and punishment systems

Interestingly, a clinical study published in “Nature Metabolism” in 2023 investigated the restorative effects of liraglutide on impaired associative learning in individuals with obesity. The study utilized a single-blind, randomized, placebo-controlled, crossover design, combined with functional magnetic resonance imaging (fMRI), to analyze the performance of 54 participants (30 with normal insulin sensitivity and 24 with impaired insulin sensitivity) on sensory associative learning tasks while receiving liraglutide and placebo treatments.^435^ Liraglutide significantly enhanced associative learning abilities in obese individuals with impaired insulin sensitivity. It modulated neural activity in the ventral striatum and midbrain pathways, affecting brain areas related to metabolic signaling.^435,436^ Liraglutide improved task performance at the behavioral level and enhanced the encoding of adaptive prediction errors, which are crucial neural signals in the learning process.^435^ (Fig. 5).

### GLP-1RAs in the cardiovascular system

In the cardiovascular system, GLP-1 can reduce cardiac ischemia-reperfusion injury through the activation of specific signaling pathways, such as the PI3K/Akt pathway.^437,438^ This involves modulating the cell apoptosis process, for example, by decreasing the Bax/Bcl-2 ratio in cardiomyocytes, reducing cytochrome C release, and caspase activation.^438,439^ GLP-1 can also enhance endothelial function by promoting the production of NO, possibly through activating eNOS, thereby affecting vasodilation, anti-inflammatory actions, and anti-atherosclerosis.^43,192,440,441^

#### GLP-1RAs and AS

AS is characterized by the formation of fibrofatty plaques within arterial walls and is one of the foremost global causes of mortality.^5^ GLP-1RAs, as exemplified by liraglutide and semaglutide, exhibit pronounced cardiovascular protective effects.^43^ Liraglutide and semaglutide have been demonstrated to be effective at reducing lipid and blood pressure levels through numerous scientific investigations, thereby contributing to the mitigation of AS and cardiovascular ailments.^442^ Preclinical studies have documented the inhibitory effects of GLP-1RAs on the development of AS in animal models.^443^ These agents exert their anti-atherosclerotic effects through various mechanisms, including by improving blood lipid profiles,^444^ preserving endothelial integrity and regulating endothelial function,^445^ as well as modulating inflammatory processes.^70^

GLP-1RAs confer substantial benefits for individuals with AS owing to their multifaceted impact on various aspects of cardiovascular health.^39,40,446^ Notably, GLP-1RAs exhibit pronounced efficacy in optimizing blood lipid profiles by effectively suppressing chylomicron secretion in the intestine, thereby mitigating the occurrence of postprandial hyperlipidemia.^447^ Moreover, these agents promote hemodynamic equilibrium,^18^ diminish thrombotic propensity,^448^ alleviate endothelial oxidative stress,^449^ attenuate inflammatory processes,^450^ and foster a favorable balance of the gut microbiota,^451^ all of which collectively contribute to the salutary effects of these agents in patients with AS.

GLP-1RAs have been shown to exert beneficial effects on the maintenance of endothelial integrity. One study suggested that GLP-1RAs exert direct endothelial protective effects by activating the GLP-1R-dependent AMPK/Akt/eNOS pathway. This pathway facilitates the generation of NO, thereby contributing to the preservation of vascular health and endothelial function. Additionally, GLP-1RAs facilitate the maintenance of endothelial barrier integrity, thus playing a crucial role in preventing vascular leakage.^452^ Endothelial cells exhibit enhanced NO production and concurrent suppression of endothelin formation, resulting in vascular smooth muscle relaxation and vasodilation mediated by the endothelium (e.g., GLP-1, exenatide, and liraglutide).^453^ Indeed, GLP-1RAs, including exenatide, liraglutide, and semaglutide, have been linked to the diminished expression of matrix metalloproteinases (MMPs).^454–456^ MMPs are a class of enzymes that play a crucial role in the breakdown of extracellular matrix components. Excessive MMP activity can lead to the weakening of fibrous caps and increase the risk of plaque rupture in atherosclerotic lesions.^454^ By reducing MMP expression, GLP-1R stimulation may help preserve the integrity of fibrous caps and mitigate the risk of plaque rupture.

GLP-1RAs have been shown to reduce the levels of systemic inflammatory markers.^457,458^ Importantly, the effective management of inflammation is widely acknowledged to play a crucial role in the prevention of cardiovascular diseases.^459,460^ The anti-inflammatory effects of GLP-1RAs contribute to the attenuation of atherosclerotic plaque lesion development through various mechanisms. First, GLP-1RAs have been shown to inhibit the expression and release of proinflammatory cytokines, such as IL-6 and TNF-α, thereby inhibiting the overall inflammatory response.^461^ GLP-1RAs can suppress the activation of nuclear factor-kappa B (NF-κB), a key transcription factor involved in the regulation of inflammatory processes.^462^ Inhibition of the NF-κB signaling pathway leads to the decreased production of inflammatory mediators, including adhesion molecules and chemokines, which are crucial for the recruitment of immune cells to sites of inflammation.^463^ In addition, liraglutide can delay the formation of AS by inducing cell cycle arrest in vascular smooth muscle cells through the AMPK pathway.^5^

#### GLP-1RAs and hypertension

**Fig. 6:**
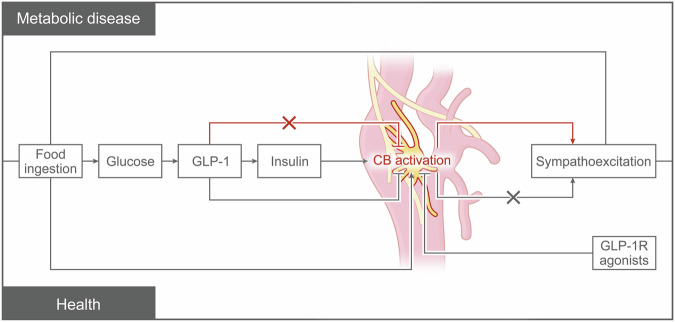
The Effects of GLP-1RAs on Carotid Body Activation. GLP-1 by inhibiting the chemoreception in carotid body cell activity to adjust the new mechanism of sympathetic nerve excitability, and points out that GLP-1 agonists can inhibit the origin of the carotid body around chemical reflection to lighten the sympathetic nerve excitability, which is expected to improve the sympathetic activity of the patients with high blood pressure, reduce blood pressure levels

The carotid body, a vital chemoreceptor in the human body, plays a pivotal role in the regulation of respiratory and cardiovascular activity, energy homeostasis, and blood glucose sensitivity.^464^ Notably, the carotid body expresses GLP-1R, which is implicated in the concurrent regulation of blood pressure and blood glucose.^465^ The downregulation of GLP-1R expression may represent a significant contributory factor to the co-occurrence of hypertension and hyperglycemia.^466^ One study proposed a novel mechanism wherein postprandial GLP-1 release, under normal physiological conditions, inhibits the activity of chemosensory cells in the carotid body, thereby counteracting sympathetic excitability mediated by elevated blood glucose or insulin levels.^465^ Impaired GLP-1 secretion or reduced GLP-1R expression may result in aberrantly heightened sympathetic excitation. However, the exogenous administration of GLP-1RAs can mitigate sympathetic excitability by suppressing the peripheral chemoreflex originating from the carotid body.^467^ Under hyperglycemic conditions, GLP-1RAs regulate hypertension by inhibiting carotid body function.^468^ Specifically, these agents can attenuate the excitability of carotid body cells and diminish sympathetic activation, ultimately leading to a reduction in sympathetic responses.^465^ This modulatory effect holds promise for ameliorating sympathetic activity in individuals with hypertension, as these agents lower blood pressure levels.^469^ Importantly, the “GLP-1-carotid body pathway” may represent a novel therapeutic target for managing cardiovascular metabolism and treating patients with diabetes and hypertension who exhibit heightened sympathetic activity.^465^ (Fig. 6).

#### GLP-1RAs and heart failure

The prevalence of left ventricular ejection fraction (LVEF)-preserved heart failure continues to increase. The symptoms in patients are severe and often accompanied by functional impairments, especially in the obese population. Heart failure with preserved ejection fraction (HFpEF) accounts for approximately half of all heart failure cases, with patients bearing a high symptomatic burden and physical limitations that impact their daily lives. These limitations include fatigue, shortness of breath, reduced exercise capacity, and limb swelling. Currently, there are no approved targeted therapies for LVEF-preserved heart failure associated with obesity. Moreover, GLP-1R has not yet been identified on human myocardial cells, negating any direct effects of GLP-1RAs on myocardial cells.^470^

Recently, studies have shown that the GLP-1RA Meg peptide also enters the ejection fraction reserve following heart failure therapy. The results of a trial of semaglutide in obese patients with HFpEF showed a significant amelioration of symptoms and improvements in motor function and weight loss compared with patients treated with a placebo.^470^ In this study, researchers randomized 529 patients with heart failure with a preserved ejection fraction and body mass index (weight in kilograms in the square of the height in meters) ≥30 to receive once-weekly injections of semaglutide (2.4 mg) or placebo for 52 weeks. The two primary endpoints were the Kansas City Cardiomyopathy Questionnaire clinical summary score (KCCQ-CSS, which ranges from 0 to 100) and the Kansas City Cardiomyopathy Questionnaire Clinical Summary Score (KCCQ-CSS). Higher scores indicate fewer symptoms and physical limitations and weight change from baseline. The confirmatory secondary endpoints included the change in the 6-min walking distance, a hierarchical composite of death and heart failure events; the difference between the change in the KCCQ-CSS and the change in the 6-min walking distance; and the change in the C-reactive protein (CRP) level.^470^ In this trial, semaglutide benefited patients with heart failure and preserved ejection fraction by intervening with upstream metabolic drivers. This agent differs from earlier therapies that were designed to reduce myocardial loading or induce neurohumoral blockade. These positive results indicate that the change in myocardial cells may not be the primary driver of HFpEF; instead, the multisystem pathologic processes that are associated with this condition have long been well established as drivers of its clinical presentation and outcomes.^470^

One could argue that the success of sodium-glucose cotransporter 2 (SGLT2) inhibitors, and now of GLP-1RAs, suggests that metabolic abnormalities play an important role in driving HFpEF.^470^ SGLT2 inhibition is also known to benefit patients with heart failure and a reduced ejection fraction.^471^ The mechanisms by which SGLT2 inhibitors treat heart failure include lowering blood glucose levels by inhibiting the SGLT2 protein in the renal tubules and reducing glucose reabsorption in the tubules.^472^ Promoting the urinary excretion of sodium and water reduces fluid retention and hypervolaemia, thereby reducing the load on the heart.^473^ It also improves myocardial energy metabolism, increases fatty acid oxidation in the myocardium, and improves myocardial energy supply.^474^ Moreover, these agents reduce the risks of myocardial inflammation and fibrosis and improve cardiac structure and function.^475^ By the way, a clinical study published in “Nature Metabolism” detailed the effects of semaglutide and dapagliflozin (an SGLT2 inhibitor) on blood sugar control in patients with T2DM of different pathophysiological types.^476^ It was found that semaglutide performed better than dapagliflozin in reducing HbA1c levels.^476^ The study results suggest that semaglutide may be a more suitable choice for patients with severe insulin deficiency, while dapagliflozin might be effective for a broader range of patients with metabolic abnormalities.^476^

If SGLT2 inhibitors and GLP-1RAs are effective in patients with heart failure (regardless of whether the ejection fraction is reduced or preserved), the commonalities between the two types of heart failure may be greater than is often thought. If so, the two types of heart failure may be very similar, except that the cause of heart failure with a preserved ejection fraction may not be the same as that with a reduced ejection fraction.^477–479^ If this is indeed the case, then both types of heart failure are syndromes caused by multiple metabolic and inflammatory changes; however, heart failure with reduced ejection fraction also has a regional cause. Therefore, intrinsic cardiac load and capacity do not improve in patients with heart failure with reduced ejection fraction, and patients HFpEF benefit only from the treatment of abnormal metabolism and inflammation.^475,480,481^

The encouraging results obtained with semaglutide in patients with heart failure and a preserved ejection fraction may provide a new option for this patient population, in which additional therapy, as well as another upstream therapy for patients with a higher BMI who have indications for this condition, is urgently needed. The clinical translation of these trial results, which will be important for the comparison of GLP-1RAs with SGLT2 inhibitors in the treatment of patients with heart failure and a preserved ejection fraction, remains to be determined.^470,478,482–484^

### GLP-1RAs in the digestive system

Treatment with GLP-1RAs can alleviate insulin signaling by reducing the phosphorylation of Akt protein (Decreased Akt-P) and activating Protein Kinase C-ε (PKC-ε).^485,486^ This affects the synthesis of triacylglycerol (TAG), phosphatidic acid (di-P PA), and diacylglycerol (DAG) in the liver.^206,487^ These changes lead to a reduction in the production of non-esterified fatty acids (LCFAs) and glucose, as well as a decrease in the synthesis of VLDL (Very Low-Density Lipoprotein).^3^

#### GLP-1RAs and NAFLD/NASH

When dietary nutrients are ingested, endogenous incretins (GIP and GLP-1) activate K and L cells in the gut, which then secrete GIP and GLP-1.^488–490^ In the pancreas, this stimulates the secretion of insulin and inhibits the secretion of glucagon.^491^ In the brain, it reduces appetite and improves satiety.^130,133^ In the gastrointestinal tract, it lowers the synthesis and secretion of triglycerides.^491^ By regulating appetite, insulin secretion, and lipid metabolism, GLP-1RAs have potential benefits in the treatment of NAFLD, NASH, and T2DM.^130,492,493^

NASH is a liver disease primarily caused by fat accumulation, which can progress to liver fibrosis, cirrhosis, or even liver cancer.^494–498^ The role of GLP-1 in NASH has garnered attention due to its potential in regulating metabolism, improving insulin sensitivity, and exerting anti-inflammatory effects.^17,499–503^ Insulin resistance, a common occurrence in NASH patients, is a key driver of the disease’s progression.^504–506^ GLP-1 enhances the insulin signaling pathway in the liver by activating the GLP-1R, especially through the phosphorylation of insulin receptor substrates and the activation of the PI3K/Akt signaling pathway, thereby increasing hepatic insulin sensitivity, facilitating glucose uptake and utilization, and reducing hepatic gluconeogenesis.^3,507^

GLP-1RAs reduce liver fat accumulation by activating AMPK, which inhibits fatty acid synthesis enzymes and promotes fatty acid β-oxidation, thus diminishing lipid droplet accumulation in hepatocytes.^508–511^ Additionally, GLP-1 mitigates liver inflammation and fibrosis by inhibiting the NF-κB pathway, reducing the release of pro-inflammatory cytokines such as TNF-α and IL-6, and thus suppressing inflammatory pathways.^307,413^

In terms of apoptosis inhibition, GLP-1 activates the PI3K/Akt signaling pathway, enhances the expression of the anti-apoptotic protein Bcl-2, and inhibits the activation of caspase family proteins, reducing cell apoptosis.^206^ This helps prevent the progression of NASH to liver fibrosis and cirrhosis. The weight-loss effect of GLP-1RAs also benefits NASH improvement, by reducing appetite and increasing energy expenditure to promote weight loss, thereby indirectly ameliorating NASH.^206,512^ Thus, GLP-1 and its agonists slow down the progression of NASH and may positively impact the reversal of liver fibrosis.^513,514^ These signaling pathways suggest that GLP-1 and its receptor agonists may influence the progression of NASH through multiple mechanisms, providing several potential therapeutic targets including: FOXO1, a transcription factor that plays a critical role in regulating glucose and lipid metabolism. GLP-1 can improve abnormalities in glucose metabolism and excessive lipid accumulation by modulating the activity of FOXO1.^5,514^ Sirt1, a protein deacetylase, plays a key role in delaying cellular aging and regulating metabolism.^515–517^ GLP-1 may improve the liver’s antioxidant capacity and metabolic function by activating Sirt1, thereby helping to alleviate the pathological changes associated with NASH.

A 2023 study published in the “Journal of Hepatology” reported on a Phase IIa trial aimed at evaluating the efficacy and safety of efinopegdutide in patients with NAFLD, comparing it to semaglutide.^144^ Efinopegdutide is a dual agonist for GLP-1R and GIPR.^144^ The study used a randomized, open-label design and employed magnetic resonance imaging technology (MRI-PDFF) to measure liver fat content (LFC) after 24 weeks of treatment. Results showed that efinopegdutide was more effective in reducing LFC compared to semaglutide. Additionally, the study assessed weight and metabolic responses, finding that efinopegdutide’s tolerability was similar to that of semaglutide, although certain gastrointestinal side effects were more common. Overall, efinopegdutide emerged as a promising option for the treatment of NAFLD.^144^

#### GLP-1RAs and gastrointestinal cancers

GLP-1 and its receptor agonists show some potential in the treatment of gastrointestinal cancers, although this remains an emerging area of research.^518–521^

##### Hepatocellular carcinoma (HCC)

Initially developed for treating diabetes, GLP-1RAs have shown potential in treating NASH, which is closely related to HCC.^513^ Studies suggest that the anti-inflammatory and metabolic effects of GLP-1RAs might also influence the progression of liver diseases, including HCC.^503,512,518^ These effects include modulating cell proliferation, inflammation, and oxidative stress in liver cells, all of which are key factors in the development and progression of HCC.^513,522^ In a mouse model induced with NASH-related HCC, treatment with liraglutide (a type of GLP-1RA) was shown to prevent the progression of hepatocellular carcinoma.^518^ This was observed through improved glycemic control, reduced occurrence of liver cancer, and better liver histology compared to the control group.^518^ Studies indicate that liraglutide may inhibit liver carcinogenesis through its metabolic effects, suggesting that GLP-1RAs could potentially play a role in preventing or managing HCC in the context of NASH.^523^ Not only hepatocellular carcinoma, but GLP-1 has also shown potential in the treatment of other gastrointestinal tumors.^524,525^

##### Pancreatic cancer

Researchers first compared the expression of GLP-1R in human pancreatic cancer tissues with adjacent non-tumorous pancreatic tissues, finding generally lower or absent expression of GLP-1R in pancreatic cancer tissues.^520^ Subsequently, the study observed that treatment with liraglutide, both in vitro (cell culture models) and in vivo (mouse models), inhibited the tumor formation and metastatic capabilities of pancreatic cancer cells by activating GLP-1R.^520^ The anti-tumor effect of liraglutide is related to its inhibition of the PI3K/Akt signaling pathway, as the activation of Akt is crucial for promoting cell survival and proliferation, and liraglutide can inhibit this process in a dose-dependent manner.^520,526^ In the context of T2DM, liraglutide, by regulating the PI3K/Akt pathway and activating GLP-1R, effectively inhibits the growth and spread of pancreatic cancer cells.^520^

##### Colorectal cancer

The potential impact of GLP-1RAs on colorectal cancer (CRC) treatment is achieved through the modulation of Bone Morphogenetic Protein 4 (BMP4).^519^ In T2DM and CRC, the regulation of BMP4 is abnormal, which is a key focus of the research.^519^ Specifically, high blood glucose-induced insulin resistance in CRC cells leads to increased BMP4 expression, which activates the BMP4-Smad1/5/8 signaling pathway.^519^ The activation of this pathway enhances cell proliferation and metastatic capabilities by promoting epithelial-mesenchymal transition (EMT), thereby increasing the invasiveness and metastatic potential of tumors. However, GLP-1RAs have been shown to reduce BMP4 levels through exogenous administration. Studies have shown that treating CRC cells with GLP-1RA can inhibit cell proliferation induced by insulin resistance by downregulating BMP4. Therefore, BMP4 becomes a potential therapeutic target in CRC, especially in a diabetic context where high blood glucose significantly affects cancer progression through the BMP4 pathway.^519^ Ultimately, GLP-1RA, by regulating BMP4 and its effects on cell proliferation and metastasis, provides a promising treatment approach.^519^ This not only demonstrates the role of GLP-1RA in diabetes management but also offers potential for integrating diabetes and cancer treatment.^520^ This finding emphasizes the importance of considering metabolic status in cancer treatment and the necessity for further research in this area.^519^ While early models have shown promising results, the application of GLP-1RAs in cancer treatment has not yet been established and requires further clinical trials.^527^

## Conclusions and perspective

Primarily recognized for their role in diabetes mellitus treatment, GLP-1RAs have demonstrated significant benefits in cardiovascular health, skeletal muscle-related diseases, obesity management, and neurodegenerative conditions, among others. In this review, we delved into the multifaceted role of GLP-1R, especially its significance in disease contexts beyond traditional glucose metabolism. It explored the mechanisms of action of GLP-1RAs and their therapeutic potential in a wide array of diseases, such as diabetes mellitus, providing new insights into metabolic disease management. These findings underscore the multifunctionality of GLP-1R as a therapeutic target and its involvement in various biological processes, emphasizing its role in addressing complex disease mechanisms. GLP-1 acts on the GLP-1R, activating multiple intracellular signaling pathways, including the cAMP/PKA pathway, the PI3K/Akt signaling pathway, and pathways related to anti-inflammatory and anti-oxidative stress responses, among others. These pathways play a crucial role in its wide-ranging therapeutic effects, extending the benefits of GLP-1RAs beyond metabolic diseases. While the therapeutic benefits of GLP-1RAs in diabetes management are well-established, their emerging role in other diseases suggests novel treatment strategies. Conclusively, research on GLP-1R and its agonists marks a promising direction in metabolic disease therapy, extending their potential beyond glucose regulation and offering hope for more comprehensive approaches in addressing metabolic diseases. This research necessitates continued exploration, potentially revolutionizing future therapeutic strategies.

In the intensely competitive GLP-1 drug market, simply enhancing drug efficacy is no longer sufficient to make new products stand out. Looking ahead, the key strategies for product innovation and differentiation are likely to focus on five main directions: expanding the therapeutic indications, achieving precision treatment with multiple biological targets, optimizing the clinical application of drug combinations, developing new formulations of oral medications, and extending the duration of drug action. These strategies not only illustrate the depth and breadth of pharmaceutical research but also signify the frontier of future medical innovations. Firstly, expanding indications is a current hotspot in GLP-1 drug research. GLP-1 drugs have been found applicable for various diseases such as cardiovascular disease, NASH, AD, and chronic kidney disease. For instance, semaglutide has been approved and is being researched for indications including obesity, T2DM, reducing cardiovascular risk, chronic kidney disease, NAFLD, and AD, indicating that the development of new indications could provide new growth opportunities for GLP-1 drugs. Secondly, efficacy is the core criterion for evaluating drugs, and currently, multi-target GLP-1 drugs have shown better efficacy. For example, triple-target agonists like retatrutide are leading multi-target GLP-1 drugs. Moreover, combination therapies have demonstrated significant advantages, such as Novo Nordisk’s CagriSema (semaglutide + cagrilintide), which shows superior glycemic control and weight reduction effects compared to semaglutide alone, demonstrating that combination therapy can achieve more than the sum of its parts while reducing side effects.^117^ Additionally, to improve patient compliance, it is crucial to extend the drug’s half-life. This has been achieved through techniques such as peptide sequence modification, peptide lipidation, albumin fusion, and Fc fusion. For example, scientists are developing GLP-1 drugs that require only monthly injections. Lastly, considering some patients’ resistance to injections, oral medications have become the preferred option. However, the oral GLP-1 product, Rybelsus, has low bioavailability and requires cumbersome daily administration, which reduces its convenience. Therefore, enhancing bioavailability and stability will be key in the future, although breakthroughs in once-weekly oral GLP-1 drugs are still awaited. With the increasing prevalence of metabolic diseases globally, interventions targeting GLP-1R could play an important role in reducing the burden of these conditions. Collaborative efforts among researchers, clinicians, and pharmaceutical developers are essential to translate these scientific insights into effective and accessible treatments.
